# Challenges for research uptake for health policymaking and practice in low- and middle-income countries: a scoping review

**DOI:** 10.1186/s12961-023-01084-5

**Published:** 2023-12-06

**Authors:** Agumasie Semahegn, Tsegahun Manyazewal, Charlotte Hanlon, Eyerusalem Getachew, Bethelhem Fekadu, Esubalew Assefa, Munir Kassa, Michael Hopkins, Tassew Woldehanna, Gail Davey, Abebaw Fekadu

**Affiliations:** 1 Centre for Innovative Drug Development and Therapeutic Trials for Africa (CDT-Africa), College of Health Sciences, Addis Ababa University, Addis Ababa, Ethiopia; 2 College of Health and Medical Sciences, Haramaya University, Harar, Ethiopia; 3 Department of Population, Family and Reproductive Health, School of Public Health, University of Ghana, Accra, Ghana; 4 Centre for Global Mental Health, Health Services and Population Research Department, King’s College London, London, UK; 5 Department of Psychiatry, WHO Collaborating Centre for Mental Health Research and Capacity-Building, School of Medicine, College of Health Sciences, Addis Ababa University, Addis Ababa, Ethiopia; 6 Health Economics and Policy Research Unit, Wolfson Institute of Population Health, Queen Mary University of London, London, UK; 7 Department of Economics, Faculty of Arts and Social Sciences, The Open University, Milton Keynes, UK; 8 Ministry of Health, Addis Ababa, Ethiopia; 9 Science Policy Research Unit, University of Sussex, Brighton, UK; 10 College of Business and Economics, Addis Ababa University, Addis Ababa, Ethiopia; 11 Global Health & Infection Department, Brighton and Sussex Medical School, Brighton, UK; 12 School of Public Health, College of Health Science, Addis Ababa University, Addis Ababa, Ethiopia

**Keywords:** Research uptake, Health policymaking, Low- and middle-income countries, Scoping review

## Abstract

**Background:**

An estimated 85% of research resources are wasted worldwide, while there is growing demand for context-based evidence-informed health policymaking. In low- and middle-income countries (LMICs), research uptake for health policymaking and practice is even lower, while little is known about the barriers to the translation of health evidence to policy and local implementation. We aimed to compile the current evidence on barriers to uptake of research in health policy and practice in LMICs using scoping review.

**Methods:**

The scoping review followed the Preferred Reporting Items for Systematic Review and Meta-Analyses-extension for Scoping Reviews (PRISMA-ScR) and the Arksey and O'Malley framework. Both published evidence and grey literature on research uptake were systematically searched from major databases (PubMed, Cochrane Library, CINAHL (EBSCO), Global Health (Ovid)) and direct Google Scholar. Literature exploring barriers to uptake of research evidence in health policy and practice in LMICs were included and their key findings were synthesized using thematic areas to address the review question.

**Results:**

A total of 4291 publications were retrieved in the initial search, of which 142 were included meeting the eligibility criteria. Overall, research uptake for policymaking and practice in LMICs was very low. The challenges to research uptake were related to lack of understanding of the local contexts, low political priority, poor stakeholder engagement and partnership, resource and capacity constraints, low system response for accountability and lack of communication and dissemination platforms.

**Conclusion:**

Important barriers to research uptake, mainly limited contextual understanding and low participation of key stakeholders and ownership, have been identified. Understanding the local research and policy context and participatory evidence production and dissemination may promote research uptake for policy and practice. Institutions that bridge the chasm between knowledge formation, evidence synthesis and translation may play critical role in the translation process.

**Supplementary Information:**

The online version contains supplementary material available at 10.1186/s12961-023-01084-5.

## Background

Globally, an estimated 85% of research resources are wasted due to errors, exaggeration and inefficiency [1, 2]. Contextual evidence synthesis is a fundamental component of evidence-informed health policymaking. Evidence synthesis relevant for the local context is needed to improve the health system’s performance and health outcomes [3]. Since the 1970s, both policymakers and researchers have given emphasis to the range of factors affecting health policymaking [4]. Evidence-informed policymaking is an interactive process that involves effective exchanges of knowledge between researchers and policymakers [5, 6]. It is aimed at minimizing policy failures in real world setting [7].

About 42% of research resource waste is avoidable through simple and inexpensive intervention [8]. However, multiple barriers exist, including poor access to good quality research, the low quantity of evidence in certain areas, lack of timeliness [9–11], information overload [12], and incompatibilities in priorities between researchers and policymakers [13]. The World Health Organization (WHO) recommends evidence-informed decision-making as having a key role in improving the effectiveness, efficiency, and equity of health policies and implementation [14]. Nevertheless, existing evidence is often not communicated in a timely way to decision-makers [3] resulting in wastage of resource invested in research [2, 15].

About 40–90% of research published from trials is not replicable [15–17], and no reliable evidence on the extent of research use, or its impact, exists [18]. These deficiencies highlight the need to create platforms for interaction between researchers and policy-makers as producers and as users of evidence [19]. This has been more apparent in the past two decades. Although evidence-informed health policymaking is central in achieving and sustaining innovative healthcare delivery in LMICs, priority-setting for health policy research remains an overlooked public health issue in LMICs [20]. There is a growing demand for platforms (centers used to create connection) dedicated to evidence uptake for policymaking both in high-income and LMICs [12, 21].

Research evidence is vital for policymaking, and the role of researchers in translating research evidence into policymaking is crucial [22]. Power relationships have significant impact on capacity development and on the links between research, policy and practice [23]. The multidisciplinary research approach has played a key role in the production of quality evidence of the complexity demand by policy [24], and has enhanced the engagement of various actors [25, 26]. Translation of evidence to policy needs collaboration among multidisciplinary scholars; collaboration between researchers and community representatives from diverse background and lay perspectives, and collaboration among community organizations across local, state, national, and international levels [24, 27, 28]. While there is some understanding of the critical barriers to research uptake and potential interventions, there is limited systematic knowledge of barriers to research uptake in LMICs. Therefore, the aim of this scoping review is to map barriers to research uptake for health policymaking in LMICs.

## Review question(s)

- What is the available evidence on barriers to uptake of research for policymaking in LMICs?
- What are the challenges to research uptake for policymaking process in LMICs?
- What recommendations might strengthen evidence uptake for policymaking in LMICs?

## Methods

### Scoping review methodology development

The scoping review followed the Preferred Reporting Items for Systematic Review and Meta-Analyses-extension for Scoping Reviews (PRISMA-ScR) [29–33] and the Arksey and O'Malley framework [34] to search published and grey literature. The completed PRISMA-ScR checklist [31] is presented as an additional file (Additional file 1).

### Search strategy for identification of relevant studies

Relevant publications on research uptake for policymaking were identified from major databases (PubMed, Cochrane library, CNAHL(EBSCO), Global Health (Ovid), and direct search of Google Scholar and other sources, including sharing of high-quality evidence via email from authors and senior researchers. Only publications since 2000 were considered due to the growing interest in evidence-informed policy making over the last two decades [21]. Literature search was performed using Medical Subject Headings (MeSH) terms that considered participant, concept and context (PCC) (Table 1). Key terms used included “health evidence” OR “health research”, OR “policy making”, OR “evidence unit” OR “evidence translation center”, “research uptake” OR “evidence-informed health policymaking” AND “challenges of research uptake” OR “barriers to research uptake” OR “barriers to evidence to policy” OR “bottlenecks to research uptake” AND “low- and middle-income countries”. The search results were imported into EndNote citation manager [35] and duplicates were removed (Additional file 2).

**Table 1:** Search terms using participant, concept and context (PCC) framework

Participants	Studies related to health research uptake for policy and program that involve stakeholders including researchers, policymakers and funders
Concept	Status of health research evidence use, evidence translation, evidence-based policymaking
	Barriers, challenges, influencing factors, bottlenecks to evidence to policy translation,
Context	Studies conducted in sub-Saharan African countries, low- and middle-income countries, developing countries, low resource settings
	Studies or literature available from January 2000 to March 2023

### Eligibility criteria

#### Inclusion criteria

- Documents written in English
- Published and relevant grey literature on evidence uptake to policy strategies
- Evidence related to research uptake challenges and opportunities in LMICs. The LMICs were selected based on the World Bank’s country classification [36].

#### Exclusion criteria

- Editorials, commentaries, fact sheets, conference abstracts and case-studies.

### Screening and selection of studies

**Fig. 1:**
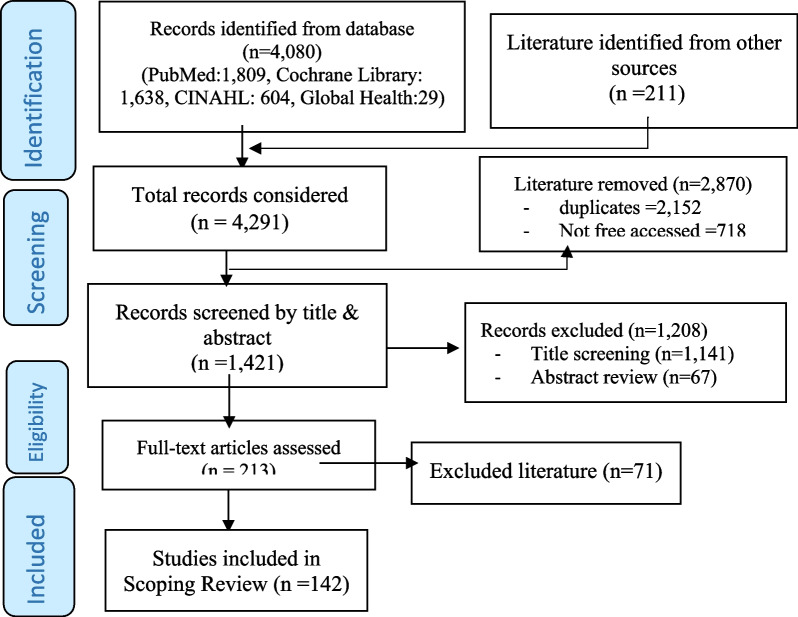
Study selection process

Publications were retrieved from selected databases, recorded using citation manager (EndNote), and duplicates were removed. Two authors (AS & EG) independently screened the title and abstracts of retrieved publications against the eligibility criteria, and based on review questions. Further appraisal assessed the methodological quality and findings before any article was included into the scoping review. Any disagreement between the two independent reviewers was resolved through consensus. If consensus was not reached, two of the senior authors (AF & TM) made a final decision based on the predetermined eligibility criteria. The screening and selection process of the scoping review was guided by the PRISMA flow diagram [29] (Fig. 1).

### Data charting and synthesis

Basic descriptions of the included studies, including authors, date, aim of the article, type of study, key findings and recommendation for action, were abstracted and recorded in a Microsoft Word table (Additional file 3). Data charting was assisted by ATLAS.ti 9 for coding of the key findings related to research uptake and barriers in line with the review questions. The codes were merged to create themes and sub-themes using an inductive approach [31, 32] of barriers to research uptake for health policymaking in LMICs. We used hybrid scoping review approach through synthesis of the key findings from included relevant documents that were guided by both PRISMA-ScR [32] and the Arksey and O'Malley framework [37]. The meta-synthesis method was used to summarize the major barriers to research uptake and key recommendations to facilitate translation of evidence to policymaking in LMICs. These included the overall situation of research uptake, barriers to research uptake and strategies used to improve research uptake.

## Results

### Available evidence on research uptake

A total of 4291 relevant documents were retrieved from studies conducted in LMICs that were grouped based on the World Bank’s country classification [36] (Fig. 1). Of these, 142 met the inclusion criteria [3, 10, 12, 38–166], and a detailed description of the documents included is presented in a table (Additional file 3). Many of the publications indicated that research uptake for health policymaking and practice is still limited in LMICs [76–88, 90, 145].

### Challenges for research uptake in LMICs

**Fig. 2:**
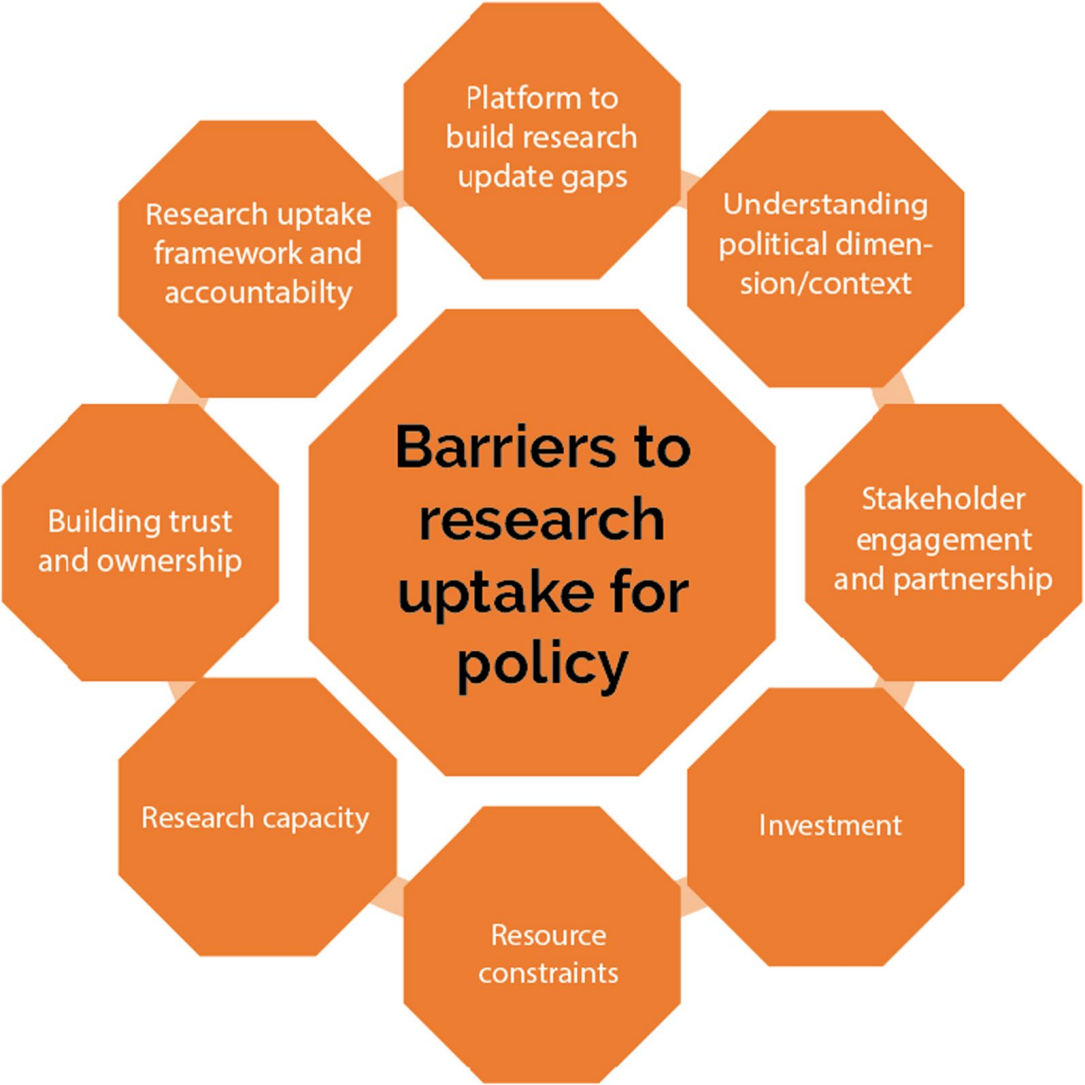
Barriers to research uptake in LMICs

The challenges of research uptake for policymaking are presented thematically. Eight main themes emerged: understanding context, stakeholder engagement and partnership, building trust and ownership, research capacity, resource constraints and misuse of resources, platform for evidence production and translation, investment in research infrastructure development, and research uptake framework and accountability (Fig. 2). Thematic narrative synthesis was performed based on the key findings.

### Understanding context

We found over 30 papers on the importance of understanding the research uptake context [49, 60, 71, 76–100, 102–122, 124, 145]. This included understanding of the political dimension and interest [3, 38, 49, 74, 75, 80, 84, 87, 91, 96, 97, 101, 106, 107, 117, 125–133, 135–138], political will [49, 60, 71, 115, 120, 139, 140] and political commitment [77, 92, 112–114, 117, 130, 138, 140–144, 146, 147], leadership [78, 85, 105, 139, 143–145, 148–150], and health policy research priority setting [38, 77–79, 83, 85, 88, 92, 106, 114, 115, 117, 138, 142, 146, 147, 151–158]. Poor understanding of the complexity of the health policymaking process, including the social and political environment [60, 71, 89, 91–97], has limited the opportunity for translation of evidence to policy. Recognizing the domains (process, content and outcome) of health policymaking is important so that useable evidence to inform policies and practices in local context is generated [79, 90, 98–100, 102, 103]. Understanding the actual context in terms of political environment will enable actual use of evidence [78, 84, 85, 108, 145], encourage institutional budgetary allocations for research [81, 86], research and policy priority setting [78, 84, 87], and support scale up for societal benefit [79, 80, 82–84, 88, 93, 104–109, 112]. Conversely, lack of credible context-specific health evidence [83, 94, 110, 111], weak local evidence, misunderstanding of decision-makers, lack of consideration of sociocultural or religious practices [60, 71, 113–117], weak involvement of advocacy coalitions, and evidence generators [118] have limited research uptake for health policymaking. Additionally, translation of evidence to policy needs mutual trust [49, 92, 119]. Lack of application of a holistic approach to evidence-based practice [113], lack of historical context [104, 120], and lack of alignment to dynamic political interests [121, 122, 124] are important contextual barriers to research uptake in LMICs. Three areas emerged from the broader theme of understanding the context of research uptake. These are political dimension, priority setting and leadership, and they are presented below.

*Political dimension*: Mapping the political dimensions and policy demand [3, 38, 49, 74, 75, 80, 84, 87, 91, 96, 97, 101, 106, 107, 117, 125–133, 135–138], understanding of political will and commitment [49, 60, 71, 77, 92, 112–115, 117, 120, 130, 138–144, 146, 147], policy interest of decisionmakers’ [38, 97, 107, 128, 130], and key policy and governance features [49, 80, 91, 131] are key drivers in evidence-informed health policymaking and implementation in LMICs. An in-depth understanding of the role of politics, how societies organize themselves in achieving collective health goals, and how different stakeholders interact in the health policymaking process [3, 74, 75, 96, 127, 128, 132, 133, 135–137] is critical in evidence translation planning. The action of policymakers may be influenced by external factors [38, 84, 97, 106, 107, 117, 128, 138] including the nature of policymaking [101], lack of political will [49, 60, 71, 115, 120, 139, 140], weak engagement of politicians [78, 143–145], and poor social service infrastructures [92, 113, 114, 140, 146, 147]. Similarly, political barriers [112], bureaucratic budget management [130], difficulty in convincing policymakers, and stakeholders [77], leadership and unclear policy direction [85, 105], all affected use of evidence. National stakeholders' perceptions, political will supporting the use of research evidence in decision-making [148], and not paying attention to structural, institutional and political condition [139, 149, 150] are critical barriers to health research use for policymaking in LMICs.

*Priority setting*: Priority setting [5, 15, 17, 22, 29–156] has been reported to have important impacts on research uptake for policymaking and practice improvement [77, 78, 83, 106, 142, 147, 151, 152, 156] and needs the involvement of key experts [106, 142, 147, 153], and policymakers [77, 78, 115, 152–154, 156] to anticipate organisational need for policymaking [83, 88, 151, 152, 156], and create a shared data administration system [38, 106]. Prioritized evidence that is aligned to ideas and actions of political priority [114, 117, 146] must be made readily available [88, 153]. Nevertheless, lack of clarity on the evidence required by policymakers in the health sector [85, 115], scarcity of dedicated units that collate research needs [85, 158] and contradiction around the scope of data needed for policymaking [79, 155] are critical barriers to health evidence translation to policy and practice in LMICs. Personal, institutional, local/national, and global priorities may compete and drive evidence translation either positively or negatively [58, 90, 115]. Stakeholders having competing priority on health research have limited efforts to address the complex health evidence translation process [78, 114, 131, 147, 159]. Policymakers' urgent needs for research evidence about health systems in LMICs have also been affected by personal financial interests, and groups competing for authority [131].

*Leadership*: Willingness and/or commitment of political leaders influence health research translation in LMICs [46, 57, 60, 71, 77–79, 81, 85, 87, 105, 109, 110, 117, 121, 128, 139, 143–145, 148–151, 154, 160, 163]^.^ Evidence-informed health policymaking relies on good leadership [92, 138, 155, 157], leaders’ willingness and commitment [46, 77, 87, 139, 151, 154, 163], decision-makers’ shared vision [57] and involvement [60, 71]. Lack of supportive, integrated and participatory leadership for research [57, 79, 81], poor political leadership with low performance [110, 121], and poor research governance [88, 139] are some of the critical leadership related challenges. Identifying and fostering public health leaders [109] will help to reduce the bureaucratic and protracted nature of policymaking and practice [128, 160]. Lack of understanding of contextual factors among key players and the powers of stakeholders [41, 42, 49, 125] was identified as important challenges to using evidence for health policymaking.

### Stakeholder engagement and partnership

Engagement of key stakeholders is a crucial strategy in translation of evidence to policy [10, 38–56, 75–77, 79–82, 84–86, 88, 90–97, 99–101, 105, 106, 108–110, 112, 114, 115, 117, 119–123, 129, 131–133, 136–141, 148–154, 156, 157, 159–166]. Lack of local stakeholder engagement is a major barrier to co-production of evidence to shape policy [40, 75, 82, 94, 109, 110, 121, 129, 131, 138, 140, 141, 149, 153, 159–162]. Translating research into policy and practice requires the intersectoral collaborative efforts of key stakeholders in LMICs [41, 54, 82, 85, 88, 112, 114, 120, 138, 149, 163]. The active engagement of funders, community organization, implementers and other stakeholders can be used to address the complex bureaucracy environment in LMICs [75, 76, 79, 82, 86, 90, 91, 109, 110, 115, 129, 132, 137, 151, 154, 160–165]. These stakeholders should be involved from the inception of project and throughout the research process. Such involvement takes into account local needs, encourages interactions, strengthens relationships, creates mutual accountability and promotes uptake of the evidence for policymaking [76, 77, 81, 85, 101, 105, 152, 163]. Likewise, evidence uptake for health policymaking needs strong public–private partnership [66, 99, 118, 123, 163], and advocacy for domestic funding, resource mobilization and collaboration [99, 163]. Stakeholder engagement is important to mitigate unmet needs [39, 91, 94, 101, 108, 122, 141, 149, 150, 166], build trust in the evidence [38, 108], and avoid duplication of efforts [38, 120, 139]. Some policymakers may not be willing to use research evidence [165]. Polarized stakeholders’ interests [117], low level of interaction between producers and users of research [43–45, 132, 141, 148], slow response to stakeholders’ requests for feedback [49, 131], and low sense of ownership [95, 106, 115, 163] led to poor research uptake for health policymaking. Relatively lower engagement of social scientists, and economists in the health research team [121, 159], and limited engagement of the media [97, 157] were also highlighted as important barriers. Poor inter-sectoral collaboration [51–53, 129, 133, 141] among public health researchers and political scientists working in international development has remained a challenge to evidence uptake.

### Building trust and ownership

Concerns about the quality of evidence have limited research uptake for policymaking [10, 12, 43, 56, 58, 59, 62, 66–68, 74–77, 81, 84, 85, 92, 94, 97, 104, 105, 108, 109, 111, 112, 116, 117, 121, 123, 127, 128, 131, 139–141, 143–145, 148, 153, 156–158]. Lack of access to good quality, timely, and relevant research outputs is a barrier to evidence uptake [10, 43, 56, 66, 67, 76, 112, 141, 156]. Lack of research literacy [56, 105], poor perceived data quality [59, 105, 156], and unavailable or inaccessible research findings [92, 156] have detrimentally influenced health evidence to policy translation in LMICs. Evidence use is limited by organizational issues, lack of robust research skills, and innovative research designs [59, 67, 97, 109, 142, 153]. Lack of locally-relevant contextual research production [12, 59, 74, 77, 116, 117, 127, 128, 140, 143, 144, 158], poor presentation of research findings [158], lack of concrete evidence on cost-effectiveness of policies [56, 139], weak institutional capacity [117, 121, 145], and low stakeholder consultation [131] cause public distrust. In addition, the beliefs and power of diverse actors [68], perceptions around the quality of existing evidence [58, 75, 77], the resistant culture of evidence users’ [58, 62, 85, 111, 123, 156], low motivation of researchers [81, 97, 104], and lack of clarity [158] are barriers to research uptake. Likewise, weak support for science-based health innovation [93], absence of effective coordination, governance and supervision [62, 105, 121, 128], inadequate integration of research into translation [105, 109, 113], expert overload, and a weak health system [63, 121] are major barriers to evidence uptake.

### Research capacity

Limited research capacity is a major challenge to research uptake for policymaking in LMICs [40, 41, 43, 49, 51, 54–56, 58–63, 66, 67, 71, 76, 77, 81, 83–88, 90–92, 97, 98, 101, 104, 105, 109–113, 115–121, 123, 130, 131, 134–140, 145–147, 150–152, 154, 155, 157, 160, 162–165]. Lack of technical competence among evidence producers and users [41, 59, 60, 71, 101, 109, 115, 121, 145, 146, 151] and lack of organizational capacity [40, 43, 47, 90, 104, 105, 117, 121, 131, 155] and inadequate infrastructure [60, 71] have significant impacts on research uptake. Untrained human resources [61, 62, 113], low research capacity to produce and use of evidence [85, 91], and limited researchers’ knowledge about research funding [81, 92, 116, 139], have all influenced health evidence to policy translation. Extensive capacity building, at the individual and organisational level [55, 60, 66, 71, 84, 85, 87, 92, 111, 118, 119, 130, 131, 150, 154, 157, 160, 164], for skilled human resource for research development [54, 55, 105, 112, 113, 120, 139, 152, 157] through in-service training [85, 86, 90, 112, 113, 146] is urgently required to improve use of research evidence. Funders need strong technical skills from researchers and integrated evidence translation [41, 110, 155]. Low levels of research understanding among evidence users have weakened research uptake for policymaking in LMICs [125, 146, 155, 157, 165].

The lack of skill that researchers have in research and translation [49, 51, 63, 66, 77, 97, 105, 134, 146], and similar skills gaps among evidence users [51, 56, 58, 62, 67, 76, 85, 98, 104, 109, 123, 146, 157], both impact research uptake in LMICs. In addition, staff shortages, high turnover [51, 60, 62, 71, 83, 91, 109], poor coordination and management, inadequate pre-service training, and insufficient specialist capacity pose challenges in translating evidence to policy. Health research uptake for policymaking and practice has suffered from low awareness, and misconceptions among evidence users [60, 62, 71, 101, 104, 112, 113, 121, 138, 139, 147]. Moreover, weak research supervision [55, 62], lack of evidence-based mentorship on new interventions [62, 81, 105, 111], and inadequate peer support in the healthcare [60, 71, 81] negatively influence evidence production and local implementation.

### Resource constraints and misuse of resources

Resource constraints are a major barrier to uptake of research for policy and practice in LMICs. [40, 46, 47, 55, 60, 61, 69–71, 79, 81, 83–85, 87, 90, 91, 97, 101, 105, 111–113, 115, 120–122, 130, 131, 138, 139, 142, 156, 158, 159, 165]. Unreliable infrastructure [111, 112, 120, 139], scarce resources and increasing numbers of patients [47, 61, 79, 101, 111–113, 120], are identified barriers to research uptake. Inadequate long-term funding for research infrastructure [79, 81, 83, 84, 91, 97, 113, 115, 121, 139, 158] and lack of local research funds [47, 69, 70, 85, 113, 115, 122, 165] are major factors affecting evidence to policy translation. Budget process bureaucracy [105, 121], corruption [105], and limited transparency [121] also influence translation efforts. Rigidity in executing research budgets [69, 91, 130, 142, 165], and use of legal proceedings [130] have also worsened evidence uptake support in LMICs.

### Platforms for evidence production and translation

Research uptake for health policy and practice requires an enabling platform for research priority setting and dissemination of findings [38, 49, 54–57, 60–62, 64, 66, 71, 77, 78, 80, 81, 84, 85, 87, 88, 90, 93–95, 105, 107, 109, 112–115, 117, 124, 128, 139, 145, 146, 148, 151, 153, 154, 156, 163], capacity strengthening and research leadership [38, 77, 85, 128]. A strong platform will facilitate engagement so that policy-makers can outline their priorities and expectations from researchers [55, 64, 77, 87, 90, 109, 154], conceptualize health research findings [80, 87, 95, 154], enhance dissemination findings [38, 80, 90, 153], networking [146], coordination [64, 85] and research utilization [66, 76, 84]. A major gap has existed in the dissemination and implementation of research findings for policy [66, 85, 90, 105, 107], in access to evidence [61, 85, 119, 128, 145, 146, 151], domestic knowledge exchange [49, 84, 113], interaction between stakeholders [66, 84, 94, 112, 113] and generating demand for evidence [139]. Inadequate health policy research infrastructure [60, 71, 78, 81, 85, 105, 109, 163], fragmentation of health information systems [95, 153, 156, 163], absence of robust institutional platforms [56, 85, 88, 114], poor access to health data [163], and lack of cost-effective technologies for health information [77, 105, 114, 117] negatively affect evidence to policy translation in LMICs. In addition, a platform for effective communication of research findings [49, 56, 66, 67, 76, 78, 82, 84, 85, 87, 97, 105, 109, 121, 128, 139, 141, 146, 150–153, 158, 160] allows policymakers to share their knowledge and experiences [78, 87, 152], and engage stakeholders [49, 139, 150, 151, 158, 160], and facilitate better packaging of key findings using plain and easy language [56, 82, 97, 146, 153] for active diffusion of innovation [109]. Limited availability of local data of the desired quality [56], researchers’ having communication and dissemination skills gaps [66, 67, 105], [49, 56, 97], poor engagement of stakeholders [66, 76, 85, 121, 128, 153], and low media use [153] are common communication barriers in LMICs.

### Investment in research infrastructure development

Research infrastructure development [43–46, 60–62, 64, 67, 71, 74, 77–79, 81, 84, 86, 88, 90, 97, 101, 105, 108, 112, 113, 119, 121, 128, 129, 149, 151, 154, 155, 157, 163, 164], including financial support to acquire essential evidence [78, 90, 105], mobilize resources for research capacity building [77, 79, 101, 112, 113, 154, 155, 164], and cooperate with national and international institutions [113] is crucial to improve research uptake. Lack of basic infrastructure for research [79, 81] and healthcare [60, 62, 64, 71, 121], widespread perceptions of unfriendly organizational environment [60, 62, 71], and structural and technical constraints between institutions [46, 61, 67, 155, 163] have negatively influenced research uptake for policy. The fact that a large proportion of health research depends on the priority setting of donors [49, 55, 59, 64, 69, 70, 77, 78, 81, 83, 85, 91–93, 97, 109, 110, 115, 117, 130, 139, 140, 142, 146, 147, 154], may not allow the uptake of evidence [78, 83, 91, 139], that might affect national health priorities. Donor research support ranged from 47 to 94% of research investment in LMICs [83]. Even though increased funding for research [81] and long-term funding for better success [55] are desired, limited time [154] affects the quality of evidence produced and its translation to policy. Donor investment in health research has fallen below that required [142, 146], and funding sustainability concerns [64, 69, 77, 92, 110, 140] will compromise research uptake for policy in LMICs.

### Evidence uptake framework and accountability

Developing a testable evidence uptake framework [38, 43, 44, 52, 62, 68, 75, 80, 82, 95, 96, 100, 102, 106, 122, 128, 136, 163] that allows in-depth integration of the fragmented body of knowledge [38, 44, 51, 62, 68, 75, 96, 100, 102, 136] will help to guide evidence uptake. System mapping to understand users’ perspectives, consult stakeholders, secure different funding streams, and design clear governance structures, leadership and staffing [82, 100] is a crucial component of effective evidence uptake. Poor information-based planning and decision-making, weak monitoring and lack of accountability and transparency [51, 62] are also barriers to effective research uptake**.** In addition, addressing bottle necks related to legal frameworks, policy, and system responses are critical to guide evidence use for policy [40, 51, 60, 62, 63, 66, 71, 72, 76, 78, 85, 86, 101, 108, 110–113, 128, 131, 139]. Enforcement and accountability of researchers and users [60, 66, 71, 76, 85, 88, 97, 108, 111, 112, 128, 131, 139] is not in place due to weak governance and regulation [60, 71, 86, 111, 113]. Though there are policy and institutional efforts to promote knowledge translation [97, 151, 155], other institutions in the health system can block these efforts, as can financial and organization pitfalls [60, 71, 90, 155], and weak national policy in the LMICs [45, 74, 84, 88, 155].

### Recommendations to strengthen research uptake in LMICs

We have identified several pieces of evidence that forward key recommendations [41, 49, 54–56, 62–67, 73, 76–78, 81, 85, 87, 88, 90, 91, 93, 97, 104–106, 108, 110, 111, 113–115, 119–121, 128, 131, 134, 146, 148, 149, 151–155, 158, 160]. We have summarized these recommendations into six key areas of action: recognize the real context, establish evidence to policy platforms, collaborate with and engage stakeholders, increase advocacy and ownership, invest in research, and build endogenous capacity.

*Evidence-to-policy platform:* Establishing knowledge translation platforms [64, 65, 77, 85, 108, 148, 151], strengthening existing platform [54, 81], and developing health research literacy programs [49, 56, 85, 97, 119, 128, 155] are crucial to enhancing the quality of evidence production and use. Strengthening institutional platforms [43, 44, 86, 88, 119, 128, 129, 157], and integrated research capacity building [64, 67, 81, 84, 108, 149] are required to improve research uptake for policy in LMICs.

*Recognize context*: Understanding political dimension and contexts [62, 63, 105, 106, 110, 121, 128, 134, 153, 160] and disseminating research findings to ensure accessibility and availability of evidence to users [56, 66, 67, 85, 112, 131] play crucial roles in improving research uptake for policymaking. Sustainable advocacy for coalition, prioritization of key players [125], addressing stakeholders’ concerns [126], and identifying opportunities and mitigating constraints [127] are also fundamental to evidence-informed health policymaking and implementation in LMICs. Balancing personal, local, institutional, and global concerns and priorities tends to lead to a sense of ownership and responsibility concerning research findings [150].

*Collaboration and stakeholders engagements*: Engagement of stakeholders is a critical step in establishing strong multisectoral collaborations and partnerships and has a key role in improving evidence uptake for policymaking [10, 48, 50, 77, 96, 99, 100, 129, 133, 136, 137, 161, 162]. Strong networking and collaboration between stakeholders [49, 56, 85, 92, 93, 101, 112], forging alliances to raise capital and investment [41, 105, 121, 149, 153], strengthening partnerships [55, 94, 117, 122], increasing community participation [95, 122, 123, 138, 157], and enhancing health innovation in LMICs [119] are robust approaches to professional transformation [85, 101], and create opportunities for transparency and communication [131, 154, 164]. Strengthening partnerships and engaging stakeholders has key role in promoting use of research findings [41, 55, 62, 73, 76, 81, 85, 90, 105, 106, 111, 113–115, 131, 146, 151, 154, 158].

*Advocacy and ownership*: Building a sense of ownership through co-production of evidence [56–59, 61–63, 84, 93, 96, 105, 106, 109, 110, 113, 115, 121, 128, 131, 148, 150, 151, 154, 155, 157–159, 164], and spanning research and policy communities [155] are essential for producing high-quality contextual evidence. Designing research translation frameworks [113, 152], global health diplomacy [111, 120] and deliberate dialogue or diplomacy efforts to mainstream research uptake for policy [76, 85, 87, 91, 120] may also make significant contribution. Similarly, research uptake advisory groups at institutional level [49, 76, 87] will facilitate in-person discussions between researchers and policymakers [42, 43, 49, 56, 61, 77, 92, 97, 124, 128, 162, 163], strengthen implementation of research uptake [85, 124, 159], and help co-production research uptake agendas [49, 56, 61, 92, 155], make better use of media, and increase links to government [42, 76, 104, 110, 120, 130].

*Invest in research:* Investments in research infrastructure to strengthen and sustain institutional research capacity [78, 91, 93, 104, 155, 158] will contribute to quality evidence generation and use in LMICs. Efficient use of scarce resources [112], mobilizing domestic funding [40, 91, 130], domestic and international resource mobilization [109, 130, 142], supporting good quality research [85, 87, 138], physical and economic infrastructure at research institutions [122], are suggested to improve evidence translation in LMICs. Co-investment with national and international funding [93] and engaging donors and policymakers in research priority setting and implementation [49, 85, 115, 117, 147] may minimize funders’ influence and achieve mutual interests. In addition, establishing a research uptake advisory board [42, 49, 56, 76, 83–85, 87, 88, 104, 108, 110, 112, 120, 122, 124, 130, 153, 155, 159, 160, 163] is important for strengthening advocacy [42, 76, 83–85, 104, 108, 110, 120, 122, 130], stakeholder engagement [108], and understanding of the local context [84]. Establishing multidisciplinary research networks [110, 157], cross-learning for researchers [56, 128, 158], and integrated knowledge translation to advance engagement [157] are important strategies to increase research uptake for policymaking.

*Endogenous capacity building*: establishing a contextual model for capacity development will help to drive evidence uptake for policymaking [40, 43, 115, 135–137, 146, 154, 162, 163]. Research and translation mentorship [55, 60, 62, 64, 65, 71, 81, 83, 105, 111, 152, 164] and supportive supervision [64, 65, 83] play key roles in identifying potential evidence for policy. Making good quality data readily available in a digestible format [59, 76, 84, 108, 148, 157] is recommended to improve research uptake in LMICs.

## Discussion

Considerable amount of evidence relevant to translation of evidence into policymaking in LMICs was identified. We found that the common barriers to research uptake are lack of understanding political dimension, low government priority, poor engagement of stakeholders, low investment in research, capacity and resource constraints, lack of ownership and trust in domestic research products, lack of a guiding research uptake framework, and lack of platform to bridge the research uptake gap in LMICs. Mapping to identify the most common forms of research use involves the direct application of research to policymaking and practice [166], and prior agenda-setting [20]. The barriers identified are consistent with the evidence, policy, and impact guiding framework of the WHO [14]. To optimize uptake of evidence in health policymaking, researchers should recognize policymakers’ priorities and prepare to engage them in long-term strategies, get their buy-ins, persuade them to act and secure a hierarchy of evidence underpinning policy [39, 167]. However, published evidence on health policy processes in LMICs is scarce, diverse, and fragmented.

We have identified common barriers limiting research uptake for health policymaking in low resource setting. Enhancing capacity for evidence-informed policy improves priority setting, filtration and amplification of evidence for policy-making and practice [20]. Institutional structures need to be improved [37, 168] through political will [169], multi-stakeholder partnership [37, 169], financial and human resources [37, 169], and evidence-based normative guidance [169]. International actors, development thinking, global partnership and networking are playing a tremendous role in research and policy. However, lack of empirical research and weak monitoring, evaluation and learning limits the impact of shaping policy with evidence in LMICs [23]. Deepening and extending health policy analysis work in LMICs requires greater levels of funding to support capacity development efforts and to generate systematic, coherent and rigorous evidence to underpin policy change [37, 170].

We identified key recommendations to improve research uptake for health policymaking in LMICs. Establishing evidence translation platforms, improving health policy research literacy and understanding the political dimension and context [171–174] are among the key recommendations to improve research uptake. Comprehensive evidence uptake approach is crucial [43, 52, 96, 128], including strong monitoring, evaluation and learning strategy of evidence to policy translation [80, 95, 102, 106, 122, 163], and evidence to policy intervention audit [62]. Likewise, engaging stakeholders from inception and /or pre-implementation [175] to dissemination is essential to understand the context of research uptake for health policymaking and practice in LMICs [38, 47, 49, 76, 80, 84, 114, 117, 121, 123, 149, 153, 160]. Establishing partnerships with global health funding organizations should prioritize the support of academic institutions’ capacity building initiatives, rigorous research, design dissemination strategies and establishing knowledge translation pathways [176].

Research uptake for health policymaking requires strategies to contextualize and balance global and local health research findings, to understand the complexity of producing high-quality research [177] and to appreciate the key role of stakeholders [177, 178] in the evidence to policymaking process [177]. Evidence producers, knowledge brokers, and end users of evidence are key actors at each phase of the research uptake process [179]. Development of context-oriented platform with the potential to facilitate research uptake for health policy making will need strong management networks and sustainable funding [37, 180]. Understanding of context [181, 182] and challenges is key to improving uptake of research for health policymaking [183]. Evidence uptake require a supportive process and mutual trust between practitioners and policy makers, and an incentive system in line with organizational vision and mission [184]. The active participation of community members and local leaders is crucial in giving opportunities to reflect their needs and interests, and to allow them to negotiate with the researcher on implementation of the study in their surroundings [173].

Stakeholder involvement will improve policy-maker confidence [58, 66], result in greater trust of local partners [94], and enhance patient and public participation [172]. An evidence to policy platform [185] for capacity strengthening [173, 186] of both evidence producers and users in implementation science will have a significant impact on research uptake [187]. Evidence to policy platform [185] play key roles in identifying effective communication strategies [188]. Systems approaches will make crucial contribution in improving efforts of translation of evidence to policy in LMICs [23, 189].

This scoping review has mainly focused on the translation of research evidence into policy. Evidence-informed practice was considered to be a manifestation of uptake; however, the evidence-to-policymaking process is far greater than evidence-informed practice or implementation.

### Implications of the findings

This scoping review identified challenges to evidence uptake and possible strategies through which this might be strengthened. A range of studies were identified, including primary studies, trials, and syntheses. Mapping the availability of quality evidence and recognizing challenges to evidence translation will enhance policymaking and practice. Dedicated centers or platforms appear likely to facilitate evidence uptake in real settings. Stakeholders in the research production and translation ecosystem should use pragmatic approaches to assess the political context and priorities, enhance collaboration, invest in research infrastructure development and adapt contextual pathways for evidence uptake. These finding will guide the focus of a Unit for Health Evidence and Policy (UHEP) which is being established to serve as a platform for evidence translation in Ethiopia and beyond. Establishing a platform to bridge the gap between researchers and policy makers is crucial to utilize available evidence for health policymaking and practice.

## Conclusion

We found substantial evidence on challenges to health research uptake for policymaking and practice in LMICs. Understanding of the national and international context and priorities, involving key stakeholders, resource and establishing a coordinating platform to facilitate capacity building, quality evidence production, communication and a framework for accountability are crucial to facilitate evidence uptake for policymaking. Barriers to poor evidence uptake for health policymaking have to be addressed through investing in research capacity building, partnership and stakeholder participation, co-mobilize resources, building trust and ownership on evidence production. This must be guided by a well-grounded theory of change framework to address barriers in LMICs. A platform for interaction and capacity building of key actors, including politicians, policy makers, academics, public health researchers and medical practitioners is essential to improve insight and establish a network for evidence sharing in LMICs.

### Supplementary Information

**Additional file1. **Completed- Preferred Reporting Items for Systematic reviews and Meta-Analyses extension for Scoping Reviews (PRISMA-ScR) Checklist.

**Additional file 2.** Literature search strategies.

**Additional file 3. **Data charting.

## Data Availability

Data can be available upon formal request to the corresponding author.

## Abbreviations

CDT-Africa: Centre for Innovative Drug Development & Therapeutic Trials for Africa
LMICs: Low- and middle-income countries
RUTAG: Research Uptake Advisory Group
UHEP: Unit for Health Evidence and Policy
WHO: World Health Organization
