# Supplementary material for: Challenges for research uptake for health policymaking and practice in low- and middle-income countries: a scoping review
Source: Health Res Policy Syst. 2023 Dec 6;21:131. doi: 10.1186/s12961-023-01084-5 (PMC10699029; doi:10.1186/s12961-023-01084-5)
Supplement: Supplementary file 2 — Additional file 2. Literature search strategies. [file 12961_2023_1084_MOESM2_ESM.pdf]

An official website of the United States government
Here's how you know

PubMed Advanced Search Builder

PubMed®

Add terms to the query box

All Fields

Enter a search term

ADD Show Index

Query box

Enter / edit your search query here

Search

History and Search Details

Search	Actions	Details	Query	Results	Time
#15	...		Search: **((Barriers OR challenges OR "influencing factors" OR bottlenecks) AND ("evidence uptake" "research uptake" OR "research evidence use" OR "evidence use" OR "evidence to policy translation" OR "research for policymaking" OR "evidence -informed policy" OR "evidence-based practice""")) AND ("low and middle income countries" OR LMICs OR "developing countries" OR "sub-Saharan Africa" OR "sub-Saharan countries" OR "low resource setting" OR "resource limited countries")**<br>("barrier"[All Fields] OR "barrier s"[All Fields] OR "barriers"[All Fields] OR ("challenge"[All Fields] OR "challenged"[All Fields] OR "challenges"[All Fields] OR "challenging"[All Fields]) OR "influencing factors"[All Fields] OR ("bottleneck"[All Fields] OR "bottlenecked"[All Fields] OR "bottlenecking"[All Fields] OR "bottlenecks"[All Fields])) AND (("evidence uptake"[All Fields] AND "research uptake"[All Fields]) OR "research evidence use"[All Fields] OR "evidence use"[All Fields] OR (("evidence"[All Fields] OR "evidences"[All Fields] OR "evident"[All Fields] OR "evidently"[All Fields]) AND ("policy"[MeSH Terms] OR "policy"[All Fields] OR "policies"[All Fields] OR "policy s"[All Fields]) AND ("translatability"[All Fields] OR "translatable"[All Fields] OR "translates"[All Fields] OR "translating"[MeSH Terms] OR "translating"[All Fields] OR "translate"[All Fields] OR "translated"[All Fields] OR "translation s"[All Fields] OR "translational"[All Fields] OR "translations"[MeSH Terms] OR "translations"[All Fields] OR "translation"[All Fields] OR "protein biosynthesis"[MeSH Terms] OR ("protein"[All Fields] AND "biosynthesis"[All Fields]) OR "protein biosynthesis"[All Fields] OR "translator"[All Fields] OR "translators"[All Fields])) OR (("research personnel"[MeSH Terms] OR ("research"[All Fields] AND "personnel"[All Fields]) OR "research personnel"[All Fields] OR "researcher"[All Fields] OR "researchers"[All Fields] OR "research"[MeSH Terms] OR "research"[All Fields] OR "research s"[All Fields] OR "researchable"[All Fields] OR "researche"[All Fields] OR "researched"[All Fields] OR "researcher s"[All	877	04:24:37

Search	Actions	Details	Query	Results	Time
			Fields] OR "researches"[All Fields] OR "researching"[All Fields] OR "researchs"[All Fields]) AND ("policymaker"[All Fields] OR "policymaker s"[All Fields] OR "policymakers"[All Fields] OR "policymaking"[All Fields])) OR "evidence informed policy"[All Fields] OR "evidence-based practice"[All Fields]) AND ("low and middle income countries"[All Fields] OR ("developing countries"[MeSH Terms] OR ("developing"[All Fields] AND "countries"[All Fields]) OR "developing countries"[All Fields] OR "lmics"[All Fields] OR "lmic s"[All Fields]) OR "developing countries"[All Fields] OR "sub-Saharan Africa"[All Fields] OR "sub-Saharan countries"[All Fields] OR "low resource setting"[All Fields] OR "resource limited countries"[All Fields])<br>**Translations**<br>**Barriers:** "barrier"[All Fields] OR "barrier's"[All Fields] OR "barriers"[All Fields]<br>**challenges:** "challenge"[All Fields] OR "challenged"[All Fields] OR "challenges"[All Fields] OR "challenging"[All Fields]<br>**bottlenecks:** "bottleneck"[All Fields] OR "bottlenecked"[All Fields] OR "bottlenecking"[All Fields] OR "bottlenecks"[All Fields]<br>**LMICs:** "developing countries"[MeSH Terms] OR ("developing"[All Fields] AND "countries"[All Fields]) OR "developing countries"[All Fields] OR "lmics"[All Fields] OR "lmic's"[All Fields]<br>**Warnings**<br>((Barriers OR challenges OR "influencing factors" OR bottlenecks) AND ("evidence uptake" "research uptake" OR "research evidence use" OR "evidence use" OR "**evidence to policy translation**" OR "**research for policymaking**" OR "evidence -informed policy" OR "evidence-based practice""")) AND ("low and middle income countries" OR LMICs OR "developing countries" OR "sub-Saharan Africa" OR "sub-Saharan countries" OR "low resource setting" OR "resource limited countries")<br>**Quoted phrases not found in phrase index:** evidence to policy translation, research for policymaking		
#14	•••		Search: **Challenges to evidence to policy translation in low and middle income countries**<br>("challenge"[All Fields] OR "challenged"[All Fields] OR "challenges"[All Fields] OR "challenging"[All Fields]) AND ("evidence"[All Fields] OR "evidences"[All Fields] OR "evident"[All Fields] OR "evidently"[All Fields]) AND ("policy"[MeSH Terms] OR "policy"[All Fields] OR "policies"[All Fields] OR "policy s"[All Fields]) AND ("translatability"[All Fields] OR "translatable"[All Fields] OR "translates"[All Fields] OR "translating"[MeSH Terms] OR "translating"[All Fields] OR "translate"[All Fields] OR "translated"[All Fields] OR "translation s"[All Fields] OR "translational"[All Fields] OR "translations"[MeSH Terms] OR "translations"[All Fields] OR "translation"[All Fields] OR "protein biosynthesis"[MeSH Terms] OR ("protein"[All Fields] AND "biosynthesis"[All Fields]) OR "protein biosynthesis"[All Fields] OR "translator"[All Fields] OR "translators"[All Fields]) AND ("developing countries"[MeSH Terms] OR ("developing"[All Fields] AND "countries"[All Fields]) OR "developing countries"[All Fields] OR ("low"[All Fields] AND "middle"[All Fields] AND "income"[All Fields] AND "countries"[All Fields]) OR "low and middle income countries"[All Fields])<br>**Translations**<br>**Challenges:** "challenge"[All Fields] OR "challenged"[All Fields] OR "challenges"[All Fields] OR "challenging"[All Fields]<br>**evidence:** "evidence"[All Fields] OR "evidences"[All Fields] OR "evident"[All Fields] OR "evidently"[All Fields]<br>**policy:** "policy"[MeSH Terms] OR "policy"[All Fields] OR "policies"[All Fields] OR "policy's"[All Fields]	119	04:16:33

Search	Actions	Details	Query	Results	Time
			**translation:** "translatability"[All Fields] OR "translatable"[All Fields] OR "translates"[All Fields] OR "translating"[MeSH Terms] OR "translating"[All Fields] OR "translate"[All Fields] OR "translated"[All Fields] OR "translation's"[All Fields] OR "translational"[All Fields] OR "translations"[MeSH Terms] OR "translations"[All Fields] OR "translation"[All Fields] OR "protein biosynthesis"[MeSH Terms] OR ("protein"[All Fields] AND "biosynthesis"[All Fields]) OR "protein biosynthesis"[All Fields] OR "translator"[All Fields] OR "translators"[All Fields]<br>**low and middle income countries:** "developing countries"[MeSH Terms] OR ("developing"[All Fields] AND "countries"[All Fields]) OR "developing countries"[All Fields] OR ("low"[All Fields] AND "middle"[All Fields] AND "income"[All Fields] AND "countries"[All Fields]) OR "low and middle income countries"[All Fields]<br>**Warnings**<br>Challenges **to** evidence **to** policy translation **in** low and middle income countries<br>**Stop words:** to, to, in		
#13	•••		Search: **Challenges to evidence uptake for health policymaking in low and middle income countries**<br>("challenge"[All Fields] OR "challenged"[All Fields] OR "challenges"[All Fields] OR "challenging"[All Fields]) AND ("evidence"[All Fields] OR "evidences"[All Fields] OR "evident"[All Fields] OR "evidently"[All Fields]) AND ("uptake"[All Fields] OR "uptakes"[All Fields] OR "uptaking"[All Fields]) AND ("health"[MeSH Terms] OR "health"[All Fields] OR "health s"[All Fields] OR "healthful"[All Fields] OR "healthfulness"[All Fields] OR "healths"[All Fields]) AND ("policymaker"[All Fields] OR "policymaker s"[All Fields] OR "policymakers"[All Fields] OR "policymaking"[All Fields]) AND ("developing countries"[MeSH Terms] OR ("developing"[All Fields] AND "countries"[All Fields]) OR "developing countries"[All Fields] OR ("low"[All Fields] AND "middle"[All Fields] AND "income"[All Fields] AND "countries"[All Fields]) OR "low and middle income countries"[All Fields])<br>**Translations**<br>**Challenges:** "challenge"[All Fields] OR "challenged"[All Fields] OR "challenges"[All Fields] OR "challenging"[All Fields]<br>**evidence:** "evidence"[All Fields] OR "evidences"[All Fields] OR "evident"[All Fields] OR "evidently"[All Fields]<br>**uptake:** "uptake"[All Fields] OR "uptakes"[All Fields] OR "uptaking"[All Fields]<br>**health:** "health"[MeSH Terms] OR "health"[All Fields] OR "health's"[All Fields] OR "healthful"[All Fields] OR "healthfulness"[All Fields] OR "healths"[All Fields]<br>**policymaking:** "policymaker"[All Fields] OR "policymaker's"[All Fields] OR "policymakers"[All Fields] OR "policymaking"[All Fields]<br>**low and middle income countries:** "developing countries"[MeSH Terms] OR ("developing"[All Fields] AND "countries"[All Fields]) OR "developing countries"[All Fields] OR ("low"[All Fields] AND "middle"[All Fields] AND "income"[All Fields] AND "countries"[All Fields]) OR "low and middle income countries"[All Fields]<br>**Warnings**<br>Challenges **to** evidence uptake **for** health policymaking **in** low and middle income countries<br>**Stop words:** to, for, in	14	04:15:56
#12	•••		Search: **Challenges to evidence use for health policymaking in sub-saharan africa countries**	71	04:14:15

Search	Actions	Details	Query	Results	Time
			("challenge"[All Fields] OR "challenged"[All Fields] OR "challenges"[All Fields] OR "challenging"[All Fields]) AND ("evidence"[All Fields] OR "evidences"[All Fields] OR "evident"[All Fields] OR "evidently"[All Fields]) AND ("health"[MeSH Terms] OR "health"[All Fields] OR "health s"[All Fields] OR "healthful"[All Fields] OR "healthfulness"[All Fields] OR "healths"[All Fields]) AND ("policymaker"[All Fields] OR "policymaker s"[All Fields] OR "policymakers"[All Fields] OR "policymaking"[All Fields]) AND ("africa south of the sahara"[MeSH Terms] OR ("africa"[All Fields] AND "south"[All Fields] AND "sahara"[All Fields]) OR "africa south of the sahara"[All Fields] OR ("sub"[All Fields] AND "saharan"[All Fields] AND "africa"[All Fields]) OR "sub saharan africa"[All Fields]) AND ("countries"[All Fields] OR "country"[All Fields] OR "country s"[All Fields] OR "countrys"[All Fields])<br><br>**Translations**<br><br>**Challenges:** "challenge"[All Fields] OR "challenged"[All Fields] OR "challenges"[All Fields] OR "challenging"[All Fields]<br>**evidence:** "evidence"[All Fields] OR "evidences"[All Fields] OR "evident"[All Fields] OR "evidently"[All Fields]<br>**health:** "health"[MeSH Terms] OR "health"[All Fields] OR "health's"[All Fields] OR "healthful"[All Fields] OR "healthfulness"[All Fields] OR "healths"[All Fields]<br>**policymaking:** "policymaker"[All Fields] OR "policymaker's"[All Fields] OR "policymakers"[All Fields] OR "policymaking"[All Fields]<br>**sub-saharan africa:** "africa south of the sahara"[MeSH Terms] OR ("africa"[All Fields] AND "south"[All Fields] AND "sahara"[All Fields]) OR "africa south of the sahara"[All Fields] OR ("sub"[All Fields] AND "saharan"[All Fields] AND "africa"[All Fields]) OR "sub saharan africa"[All Fields]<br>**countries:** "countries"[All Fields] OR "country"[All Fields] OR "country's"[All Fields] OR "countrys"[All Fields]<br><br>**Warnings**<br><br>Challenges **to** evidence **use for** health policymaking **in** sub-saharan africa countries<br><br>**Stop words:** to, use, for, in		
#11	•••		Search: **barriers to evidence use for health policymaking in sub-saharan africa countries**<br><br>("barrier"[All Fields] OR "barrier s"[All Fields] OR "barriers"[All Fields]) AND ("evidence"[All Fields] OR "evidences"[All Fields] OR "evident"[All Fields] OR "evidently"[All Fields]) AND ("health"[MeSH Terms] OR "health"[All Fields] OR "health s"[All Fields] OR "healthful"[All Fields] OR "healthfulness"[All Fields] OR "healths"[All Fields]) AND ("policymaker"[All Fields] OR "policymaker s"[All Fields] OR "policymakers"[All Fields] OR "policymaking"[All Fields]) AND ("africa south of the sahara"[MeSH Terms] OR ("africa"[All Fields] AND "south"[All Fields] AND "sahara"[All Fields]) OR "africa south of the sahara"[All Fields] OR ("sub"[All Fields] AND "saharan"[All Fields] AND "africa"[All Fields]) OR "sub saharan africa"[All Fields]) AND ("countries"[All Fields] OR "country"[All Fields] OR "country s"[All Fields] OR "countrys"[All Fields])<br><br>**Translations**<br><br>**barriers:** "barrier"[All Fields] OR "barrier's"[All Fields] OR "barriers"[All Fields]<br>**evidence:** "evidence"[All Fields] OR "evidences"[All Fields] OR "evident"[All Fields] OR "evidently"[All Fields]<br>**health:** "health"[MeSH Terms] OR "health"[All Fields] OR "health's"[All Fields] OR "healthful"[All Fields] OR "healthfulness"[All Fields] OR "healths"[All Fields]<br>**policymaking:** "policymaker"[All Fields] OR "policymaker's"[All Fields] OR "policymakers"[All Fields] OR "policymaking"[All Fields]	47	03:49:38

Search	Actions	Details	Query	Results	Time
			**sub-saharan africa:** "africa south of the sahara"[MeSH Terms] OR ("africa"[All Fields] AND "south"[All Fields] AND "sahara"[All Fields]) OR "africa south of the sahara"[All Fields] OR ("sub"[All Fields] AND "saharan"[All Fields] AND "africa"[All Fields]) OR "sub saharan africa"[All Fields]<br>**countries:** "countries"[All Fields] OR "country"[All Fields] OR "country's"[All Fields] OR "countrys"[All Fields]<br><br>**Warnings**<br><br>barriers **to** evidence **use for** health policymaking **in** sub-saharan africa countries<br><br>**Stop words:** to, use, for, in		
#10	•••		Search: **barriers to research evidence uptake for health policy making in sub saharan africa countries**<br><br>("barrier"[All Fields] OR "barrier s"[All Fields] OR "barriers"[All Fields]) AND ("research personnel"[MeSH Terms] OR ("research"[All Fields] AND "personnel"[All Fields]) OR "research personnel"[All Fields] OR "researcher"[All Fields] OR "researchers"[All Fields] OR "research"[MeSH Terms] OR "research"[All Fields] OR "research s"[All Fields] OR "researchable"[All Fields] OR "researche"[All Fields] OR "researched"[All Fields] OR "researcher s"[All Fields] OR "researches"[All Fields] OR "researching"[All Fields] OR "researchs"[All Fields]) AND ("evidence"[All Fields] OR "evidences"[All Fields] OR "evident"[All Fields] OR "evidently"[All Fields]) AND ("uptake"[All Fields] OR "uptakes"[All Fields] OR "uptaking"[All Fields]) AND ("health policy"[MeSH Terms] OR ("health"[All Fields] AND "policy"[All Fields]) OR "health policy"[All Fields]) AND ("makes"[All Fields] OR "making"[All Fields] OR "makings"[All Fields]) AND ("africa south of the sahara"[MeSH Terms] OR ("africa"[All Fields] AND "south"[All Fields] AND "sahara"[All Fields]) OR "africa south of the sahara"[All Fields] OR ("sub"[All Fields] AND "saharan"[All Fields] AND "africa"[All Fields]) OR "sub saharan africa"[All Fields]) AND ("countries"[All Fields] OR "country"[All Fields] OR "country s"[All Fields] OR "countrys"[All Fields])<br><br>**Translations**<br><br>**barriers:** "barrier"[All Fields] OR "barrier's"[All Fields] OR "barriers"[All Fields]<br>**research:** "research personnel"[MeSH Terms] OR ("research"[All Fields] AND "personnel"[All Fields]) OR "research personnel"[All Fields] OR "researcher"[All Fields] OR "researchers"[All Fields] OR "research"[MeSH Terms] OR "research"[All Fields] OR "research's"[All Fields] OR "researchable"[All Fields] OR "researche"[All Fields] OR "researched"[All Fields] OR "researcher's"[All Fields] OR "researches"[All Fields] OR "researching"[All Fields] OR "researchs"[All Fields]<br>**evidence:** "evidence"[All Fields] OR "evidences"[All Fields] OR "evident"[All Fields] OR "evidently"[All Fields]<br>**uptake:** "uptake"[All Fields] OR "uptakes"[All Fields] OR "uptaking"[All Fields]<br>**health policy:** "health policy"[MeSH Terms] OR ("health"[All Fields] AND "policy"[All Fields]) OR "health policy"[All Fields]<br>**making:** "makes"[All Fields] OR "making"[All Fields] OR "makings"[All Fields]<br>**sub saharan africa:** "africa south of the sahara"[MeSH Terms] OR ("africa"[All Fields] AND "south"[All Fields] AND "sahara"[All Fields]) OR "africa south of the sahara"[All Fields] OR ("sub"[All Fields] AND "saharan"[All Fields] AND "africa"[All Fields]) OR "sub saharan africa"[All Fields]<br>**countries:** "countries"[All Fields] OR "country"[All Fields] OR "country's"[All Fields] OR "countrys"[All Fields]<br><br>**Warnings**	7	03:48:02

Search	Actions	Details	Query	Results	Time
			barriers **to** research evidence uptake **for** health policy making **in** sub saharan africa countries<br>**Stop words:** to, for, in		
#9	•••		Search: **Barriers to research evidence uptake for health policymaking in sub-Saharan Africa countries**<br>("barrier"[All Fields] OR "barrier s"[All Fields] OR "barriers"[All Fields]) AND ("research personnel"[MeSH Terms] OR ("research"[All Fields] AND "personnel"[All Fields]) OR "research personnel"[All Fields] OR "researcher"[All Fields] OR "researchers"[All Fields] OR "research"[MeSH Terms] OR "research"[All Fields] OR "research s"[All Fields] OR "researchable"[All Fields] OR "researche"[All Fields] OR "researched"[All Fields] OR "researcher s"[All Fields] OR "researches"[All Fields] OR "researching"[All Fields] OR "researchs"[All Fields]) AND ("evidence"[All Fields] OR "evidences"[All Fields] OR "evident"[All Fields] OR "evidently"[All Fields]) AND ("uptake"[All Fields] OR "uptakes"[All Fields] OR "uptaking"[All Fields]) AND ("health"[MeSH Terms] OR "health"[All Fields] OR "health s"[All Fields] OR "healthful"[All Fields] OR "healthfulness"[All Fields] OR "healths"[All Fields]) AND ("policymaker"[All Fields] OR "policymaker s"[All Fields] OR "policymakers"[All Fields] OR "policymaking"[All Fields]) AND ("africa south of the sahara"[MeSH Terms] OR ("africa"[All Fields] AND "south"[All Fields] AND "sahara"[All Fields]) OR "africa south of the sahara"[All Fields] OR ("sub"[All Fields] AND "saharan"[All Fields] AND "africa"[All Fields]) OR "sub saharan africa"[All Fields]) AND ("countries"[All Fields] OR "country"[All Fields] OR "country s"[All Fields] OR "countrys"[All Fields])<br>**Translations**<br>**Barriers:** "barrier"[All Fields] OR "barrier's"[All Fields] OR "barriers"[All Fields]<br>**research:** "research personnel"[MeSH Terms] OR ("research"[All Fields] AND "personnel"[All Fields]) OR "research personnel"[All Fields] OR "researcher"[All Fields] OR "researchers"[All Fields] OR "research"[MeSH Terms] OR "research"[All Fields] OR "research's"[All Fields] OR "researchable"[All Fields] OR "researche"[All Fields] OR "researched"[All Fields] OR "researcher's"[All Fields] OR "researches"[All Fields] OR "researching"[All Fields] OR "researchs"[All Fields]<br>**evidence:** "evidence"[All Fields] OR "evidences"[All Fields] OR "evident"[All Fields] OR "evidently"[All Fields]<br>**uptake:** "uptake"[All Fields] OR "uptakes"[All Fields] OR "uptaking"[All Fields]<br>**health:** "health"[MeSH Terms] OR "health"[All Fields] OR "health's"[All Fields] OR "healthful"[All Fields] OR "healthfulness"[All Fields] OR "healths"[All Fields]<br>**policymaking:** "policymaker"[All Fields] OR "policymaker's"[All Fields] OR "policymakers"[All Fields] OR "policymaking"[All Fields]<br>**sub-Saharan Africa:** "africa south of the sahara"[MeSH Terms] OR ("africa"[All Fields] AND "south"[All Fields] AND "sahara"[All Fields]) OR "africa south of the sahara"[All Fields] OR ("sub"[All Fields] AND "saharan"[All Fields] AND "africa"[All Fields]) OR "sub saharan africa"[All Fields]<br>**countries:** "countries"[All Fields] OR "country"[All Fields] OR "country's"[All Fields] OR "countrys"[All Fields]<br>**Warnings**<br>Barriers **to** research evidence uptake **for** health policymaking **in** sub-Saharan Africa countries<br>**Stop words:** to, for, in	12	03:47:59
#8	•••		Search: **Barriers to research evidence to policy translation in sub-Saharan Africa countries**	25	03:46:17

Search	Actions	Details	Query	Results	Time

("barrier"[All Fields] OR "barrier s"[All Fields] OR "barriers"[All Fields]) AND ("research personnel"[MeSH Terms] OR ("research"[All Fields] AND "personnel"[All Fields]) OR "research personnel"[All Fields] OR "researcher"[All Fields] OR "researchers"[All Fields] OR "research"[MeSH Terms] OR "research"[All Fields] OR "research s"[All Fields] OR "researchable"[All Fields] OR "researche"[All Fields] OR "researched"[All Fields] OR "researcher s"[All Fields] OR "researches"[All Fields] OR "researching"[All Fields] OR "researchs"[All Fields]) AND ("evidence"[All Fields] OR "evidences"[All Fields] OR "evident"[All Fields] OR "evidently"[All Fields]) AND ("policy"[MeSH Terms] OR "policy"[All Fields] OR "policies"[All Fields] OR "policy s"[All Fields]) AND ("translatability"[All Fields] OR "translatable"[All Fields] OR "translates"[All Fields] OR "translating"[MeSH Terms] OR "translating"[All Fields] OR "translate"[All Fields] OR "translated"[All Fields] OR "translation s"[All Fields] OR "translational"[All Fields] OR "translations"[MeSH Terms] OR "translations"[All Fields] OR "translation"[All Fields] OR "protein biosynthesis"[MeSH Terms] OR ("protein"[All Fields] AND "biosynthesis"[All Fields]) OR "protein biosynthesis"[All Fields] OR "translator"[All Fields] OR "translators"[All Fields]) AND ("africa south of the sahara"[MeSH Terms] OR ("africa"[All Fields] AND "south"[All Fields] AND "sahara"[All Fields]) OR "africa south of the sahara"[All Fields] OR ("sub"[All Fields] AND "saharan"[All Fields] AND "africa"[All Fields]) OR "sub saharan africa"[All Fields]) AND ("countries"[All Fields] OR "country"[All Fields] OR "country s"[All Fields] OR "countrys"[All Fields])

Translations

Barriers: "barrier"[All Fields] OR "barrier's"[All Fields] OR "barriers"[All Fields]
research: "research personnel"[MeSH Terms] OR ("research"[All Fields] AND "personnel"[All Fields]) OR "research personnel"[All Fields] OR "researcher"[All Fields] OR "researchers"[All Fields] OR "research"[MeSH Terms] OR "research"[All Fields] OR "research's"[All Fields] OR "researchable"[All Fields] OR "researche"[All Fields] OR "researched"[All Fields] OR "researcher's"[All Fields] OR "researches"[All Fields] OR "researching"[All Fields] OR "researchs"[All Fields]
evidence: "evidence"[All Fields] OR "evidences"[All Fields] OR "evident"[All Fields] OR "evidently"[All Fields]
policy: "policy"[MeSH Terms] OR "policy"[All Fields] OR "policies"[All Fields] OR "policy's"[All Fields]
translation: "translatability"[All Fields] OR "translatable"[All Fields] OR "translates"[All Fields] OR "translating"[MeSH Terms] OR "translating"[All Fields] OR "translate"[All Fields] OR "translated"[All Fields] OR "translation's"[All Fields] OR "translational"[All Fields] OR "translations"[MeSH Terms] OR "translations"[All Fields] OR "translation"[All Fields] OR "protein biosynthesis"[MeSH Terms] OR ("protein"[All Fields] AND "biosynthesis"[All Fields]) OR "protein biosynthesis"[All Fields] OR "translator"[All Fields] OR "translators"[All Fields]
sub-Saharan Africa: "africa south of the sahara"[MeSH Terms] OR ("africa"[All Fields] AND "south"[All Fields] AND "sahara"[All Fields]) OR "africa south of the sahara"[All Fields] OR ("sub"[All Fields] AND "saharan"[All Fields] AND "africa"[All Fields]) OR "sub saharan africa"[All Fields]
countries: "countries"[All Fields] OR "country"[All Fields] OR "country's"[All Fields] OR "countrys"[All Fields]

Warnings

Barriers **to** research evidence **to** policy translation **in** sub-Saharan Africa countries

Stop words: to, to, in

Search	Actions	Details	Query	Results	Time
#7	•••		Search: **Barriers for research evidence to policy translation in low and middle income countries**<br>("barrier"[All Fields] OR "barrier s"[All Fields] OR "barriers"[All Fields]) AND ("research personnel"[MeSH Terms] OR ("research"[All Fields] AND "personnel"[All Fields]) OR "research personnel"[All Fields] OR "researcher"[All Fields] OR "researchers"[All Fields] OR "research"[MeSH Terms] OR "research"[All Fields] OR "research s"[All Fields] OR "researchable"[All Fields] OR "researche"[All Fields] OR "researched"[All Fields] OR "researcher s"[All Fields] OR "researches"[All Fields] OR "researching"[All Fields] OR "researchs"[All Fields]) AND ("evidence"[All Fields] OR "evidences"[All Fields] OR "evident"[All Fields] OR "evidently"[All Fields]) AND ("policy"[MeSH Terms] OR "policy"[All Fields] OR "policies"[All Fields] OR "policy s"[All Fields]) AND ("translatability"[All Fields] OR "translatable"[All Fields] OR "translates"[All Fields] OR "translating"[MeSH Terms] OR "translating"[All Fields] OR "translate"[All Fields] OR "translated"[All Fields] OR "translation s"[All Fields] OR "translational"[All Fields] OR "translations"[MeSH Terms] OR "translations"[All Fields] OR "translation"[All Fields] OR "protein biosynthesis"[MeSH Terms] OR ("protein"[All Fields] AND "biosynthesis"[All Fields]) OR "protein biosynthesis"[All Fields] OR "translator"[All Fields] OR "translators"[All Fields]) AND ("developing countries"[MeSH Terms] OR ("developing"[All Fields] AND "countries"[All Fields]) OR "developing countries"[All Fields] OR ("low"[All Fields] AND "middle"[All Fields] AND "income"[All Fields] AND "countries"[All Fields]) OR "low and middle income countries"[All Fields])<br>**Translations**<br>**Barriers:** "barrier"[All Fields] OR "barrier's"[All Fields] OR "barriers"[All Fields]<br>**research:** "research personnel"[MeSH Terms] OR ("research"[All Fields] AND "personnel"[All Fields]) OR "research personnel"[All Fields] OR "researcher"[All Fields] OR "researchers"[All Fields] OR "research"[MeSH Terms] OR "research"[All Fields] OR "research's"[All Fields] OR "researchable"[All Fields] OR "researche"[All Fields] OR "researched"[All Fields] OR "researcher's"[All Fields] OR "researches"[All Fields] OR "researching"[All Fields] OR "researchs"[All Fields]<br>**evidence:** "evidence"[All Fields] OR "evidences"[All Fields] OR "evident"[All Fields] OR "evidently"[All Fields]<br>**policy:** "policy"[MeSH Terms] OR "policy"[All Fields] OR "policies"[All Fields] OR "policy's"[All Fields]<br>**translation:** "translatability"[All Fields] OR "translatable"[All Fields] OR "translates"[All Fields] OR "translating"[MeSH Terms] OR "translating"[All Fields] OR "translate"[All Fields] OR "translated"[All Fields] OR "translation's"[All Fields] OR "translational"[All Fields] OR "translations"[MeSH Terms] OR "translations"[All Fields] OR "translation"[All Fields] OR "protein biosynthesis"[MeSH Terms] OR ("protein"[All Fields] AND "biosynthesis"[All Fields]) OR "protein biosynthesis"[All Fields] OR "translator"[All Fields] OR "translators"[All Fields]<br>**low and middle income countries:** "developing countries"[MeSH Terms] OR ("developing"[All Fields] AND "countries"[All Fields]) OR "developing countries"[All Fields] OR ("low"[All Fields] AND "middle"[All Fields] AND "income"[All Fields] AND "countries"[All Fields]) OR "low and middle income countries"[All Fields]<br>**Warnings**<br>Barriers **for** research evidence **to** policy translation **in** low and middle income countries<br>**Stop words:** for, to, in	64	03:45:53

Search	Actions	Details	Query	Results	Time
#6	•••		Search: **Barriers to research evidence to policy translation in low and middle income countries**	64	03:44:39
#5	•••		Search: **Barriers to research uptake for health policy making in low and middle income countries**<br>("barrier"[All Fields] OR "barrier s"[All Fields] OR "barriers"[All Fields]) AND ("research personnel"[MeSH Terms] OR ("research"[All Fields] AND "personnel"[All Fields]) OR "research personnel"[All Fields] OR "researcher"[All Fields] OR "researchers"[All Fields] OR "research"[MeSH Terms] OR "research"[All Fields] OR "research s"[All Fields] OR "researchable"[All Fields] OR "researche"[All Fields] OR "researched"[All Fields] OR "researcher s"[All Fields] OR "researches"[All Fields] OR "researching"[All Fields] OR "researchs"[All Fields]) AND ("uptake"[All Fields] OR "uptakes"[All Fields] OR "uptaking"[All Fields]) AND ("health policy"[MeSH Terms] OR ("health"[All Fields] AND "policy"[All Fields]) OR "health policy"[All Fields]) AND ("makes"[All Fields] OR "making"[All Fields] OR "makings"[All Fields]) AND ("developing countries"[MeSH Terms] OR ("developing"[All Fields] AND "countries"[All Fields]) OR "developing countries"[All Fields] OR ("low"[All Fields] AND "middle"[All Fields] AND "income"[All Fields] AND "countries"[All Fields]) OR "low and middle income countries"[All Fields])<br><br>**Translations**<br><br>**Barriers:** "barrier"[All Fields] OR "barrier's"[All Fields] OR "barriers"[All Fields]<br>**research:** "research personnel"[MeSH Terms] OR ("research"[All Fields] AND "personnel"[All Fields]) OR "research personnel"[All Fields] OR "researcher"[All Fields] OR "researchers"[All Fields] OR "research"[MeSH Terms] OR "research"[All Fields] OR "research's"[All Fields] OR "researchable"[All Fields] OR "researche"[All Fields] OR "researched"[All Fields] OR "researcher's"[All Fields] OR "researches"[All Fields] OR "researching"[All Fields] OR "researchs"[All Fields]<br>**uptake:** "uptake"[All Fields] OR "uptakes"[All Fields] OR "uptaking"[All Fields]<br>**health policy:** "health policy"[MeSH Terms] OR ("health"[All Fields] AND "policy"[All Fields]) OR "health policy"[All Fields]<br>**making:** "makes"[All Fields] OR "making"[All Fields] OR "makings"[All Fields]<br>**low and middle income countries:** "developing countries"[MeSH Terms] OR ("developing"[All Fields] AND "countries"[All Fields]) OR "developing countries"[All Fields] OR ("low"[All Fields] AND "middle"[All Fields] AND "income"[All Fields] AND "countries"[All Fields]) OR "low and middle income countries"[All Fields]<br><br>**Warnings**<br><br>Barriers **to** research uptake **for** health policy making **in** low and middle income countries<br><br>**Stop words:** to, for, in	18	03:42:48
#4	•••		Search: **research uptake for health policy making in low and middle income countries**<br>("research personnel"[MeSH Terms] OR ("research"[All Fields] AND "personnel"[All Fields]) OR "research personnel"[All Fields] OR "researcher"[All Fields] OR "researchers"[All Fields] OR "research"[MeSH Terms] OR "research"[All Fields] OR "research s"[All Fields] OR "researchable"[All Fields] OR "researche"[All Fields] OR "researched"[All Fields] OR "researcher s"[All Fields] OR "researches"[All Fields] OR "researching"[All Fields] OR "researchs"[All Fields]) AND ("uptake"[All Fields] OR "uptakes"[All Fields] OR "uptaking"[All Fields]) AND ("health policy"[MeSH Terms] OR ("health"[All Fields] AND "policy"[All Fields]) OR	81	03:38:20

Search	Actions	Details	Query	Results	Time
			"health policy"[All Fields]) AND ("makes"[All Fields] OR "making"[All Fields] OR "makings"[All Fields]) AND ("developing countries"[MeSH Terms] OR ("developing"[All Fields] AND "countries"[All Fields]) OR "developing countries"[All Fields] OR ("low"[All Fields] AND "middle"[All Fields] AND "income"[All Fields] AND "countries"[All Fields]) OR "low and middle income countries"[All Fields])<br><br>**Translations**<br><br>**research:** "research personnel"[MeSH Terms] OR ("research"[All Fields] AND "personnel"[All Fields]) OR "research personnel"[All Fields] OR "researcher"[All Fields] OR "researchers"[All Fields] OR "research"[MeSH Terms] OR "research"[All Fields] OR "research's"[All Fields] OR "researchable"[All Fields] OR "researche"[All Fields] OR "researched"[All Fields] OR "researcher's"[All Fields] OR "researches"[All Fields] OR "researching"[All Fields] OR "researchs"[All Fields]<br>**uptake:** "uptake"[All Fields] OR "uptakes"[All Fields] OR "uptaking"[All Fields]<br>**health policy:** "health policy"[MeSH Terms] OR ("health"[All Fields] AND "policy"[All Fields]) OR "health policy"[All Fields]<br>**making:** "makes"[All Fields] OR "making"[All Fields] OR "makings"[All Fields]<br>**low and middle income countries:** "developing countries"[MeSH Terms] OR ("developing"[All Fields] AND "countries"[All Fields]) OR "developing countries"[All Fields] OR ("low"[All Fields] AND "middle"[All Fields] AND "income"[All Fields] AND "countries"[All Fields]) OR "low and middle income countries"[All Fields]<br><br>**Warnings**<br><br>research uptake **for** health policy making **in** low and middle income countries<br><br>**Stop words:** for, in		
#3	•••		Search: **research uptake for health policymaking in low and middle income countries**<br><br>("research personnel"[MeSH Terms] OR ("research"[All Fields] AND "personnel"[All Fields]) OR "research personnel"[All Fields] OR "researcher"[All Fields] OR "researchers"[All Fields] OR "research"[MeSH Terms] OR "research"[All Fields] OR "research s"[All Fields] OR "researchable"[All Fields] OR "researche"[All Fields] OR "researched"[All Fields] OR "researcher s"[All Fields] OR "researches"[All Fields] OR "researching"[All Fields] OR "researchs"[All Fields]) AND ("uptake"[All Fields] OR "uptakes"[All Fields] OR "uptaking"[All Fields]) AND ("health"[MeSH Terms] OR "health"[All Fields] OR "health s"[All Fields] OR "healthful"[All Fields] OR "healthfulness"[All Fields] OR "healths"[All Fields]) AND ("policymaker"[All Fields] OR "policymaker s"[All Fields] OR "policymakers"[All Fields] OR "policymaking"[All Fields]) AND ("developing countries"[MeSH Terms] OR ("developing"[All Fields] AND "countries"[All Fields]) OR "developing countries"[All Fields] OR ("low"[All Fields] AND "middle"[All Fields] AND "income"[All Fields] AND "countries"[All Fields]) OR "low and middle income countries"[All Fields])<br><br>**Translations**<br><br>**research:** "research personnel"[MeSH Terms] OR ("research"[All Fields] AND "personnel"[All Fields]) OR "research personnel"[All Fields] OR "researcher"[All Fields] OR "researchers"[All Fields] OR "research"[MeSH Terms] OR "research"[All Fields] OR "research's"[All Fields] OR "researchable"[All Fields] OR "researche"[All Fields] OR "researched"[All Fields] OR "researcher's"[All Fields] OR "researches"[All Fields] OR "researching"[All Fields] OR "researchs"[All Fields]	78	03:05:12

Search	Actions	Details	Query	Results	Time
			**uptake:** "uptake"[All Fields] OR "uptakes"[All Fields] OR "uptaking"[All Fields]<br>**health:** "health"[MeSH Terms] OR "health"[All Fields] OR "health's"[All Fields] OR "healthful"[All Fields] OR "healthfulness"[All Fields] OR "healths"[All Fields]<br>**policymaking:** "policymaker"[All Fields] OR "policymaker's"[All Fields] OR "policymakers"[All Fields] OR "policymaking"[All Fields]<br>**low and middle income countries:** "developing countries"[MeSH Terms] OR ("developing"[All Fields] AND "countries"[All Fields]) OR "developing countries"[All Fields] OR ("low"[All Fields] AND "middle"[All Fields] AND "income"[All Fields] AND "countries"[All Fields]) OR "low and middle income countries"[All Fields]<br>**Warnings**<br>research uptake **for** health policymaking **in** low and middle income countries<br>**Stop words:** for, in		
#1	•••		Search: **barriers to research uptake in low income countries**<br>("barrier"[All Fields] OR "barrier s"[All Fields] OR "barriers"[All Fields]) AND ("research personnel"[MeSH Terms] OR ("research"[All Fields] AND "personnel"[All Fields]) OR "research personnel"[All Fields] OR "researcher"[All Fields] OR "researchers"[All Fields] OR "research"[MeSH Terms] OR "research"[All Fields] OR "research s"[All Fields] OR "researchable"[All Fields] OR "researche"[All Fields] OR "researched"[All Fields] OR "researcher s"[All Fields] OR "researches"[All Fields] OR "researching"[All Fields] OR "researchs"[All Fields]) AND ("uptake"[All Fields] OR "uptakes"[All Fields] OR "uptaking"[All Fields]) AND ("developing countries"[MeSH Terms] OR ("developing"[All Fields] AND "countries"[All Fields]) OR "developing countries"[All Fields] OR ("low"[All Fields] AND "income"[All Fields] AND "countries"[All Fields]) OR "low income countries"[All Fields])<br>**Translations**<br>**barriers:** "barrier"[All Fields] OR "barrier's"[All Fields] OR "barriers"[All Fields]<br>**research:** "research personnel"[MeSH Terms] OR ("research"[All Fields] AND "personnel"[All Fields]) OR "research personnel"[All Fields] OR "researcher"[All Fields] OR "researchers"[All Fields] OR "research"[MeSH Terms] OR "research"[All Fields] OR "research's"[All Fields] OR "researchable"[All Fields] OR "researche"[All Fields] OR "researched"[All Fields] OR "researcher's"[All Fields] OR "researches"[All Fields] OR "researching"[All Fields] OR "researchs"[All Fields]<br>**uptake:** "uptake"[All Fields] OR "uptakes"[All Fields] OR "uptaking"[All Fields]<br>**low income countries:** "developing countries"[MeSH Terms] OR ("developing"[All Fields] AND "countries"[All Fields]) OR "developing countries"[All Fields] OR ("low"[All Fields] AND "income"[All Fields] AND "countries"[All Fields]) OR "low income countries"[All Fields]<br>**Warnings**<br>barriers **to** research uptake **in** low income countries<br>**Stop words:** to, in	381	03:04:13
#2	•••		Search: **Barriers to evidence uptake for health policymaking in low- and middle-income countries**<br>("barrier"[All Fields] OR "barrier s"[All Fields] OR "barriers"[All Fields]) AND ("evidence"[All Fields] OR "evidences"[All Fields] OR "evident"[All Fields] OR "evidently"[All Fields]) AND ("uptake"[All Fields] OR "uptakes"[All Fields] OR "uptaking"[All Fields]) AND ("health"[MeSH Terms] OR "health"	20	02:01:07

Search	Actions	Details	Query	Results	Time
			[All Fields] OR "health s"[All Fields] OR "healthful"[All Fields] OR "healthfulness"[All Fields] OR "healths"[All Fields]) AND ("policymaker"[All Fields] OR "policymaker s"[All Fields] OR "policymakers"[All Fields] OR "policymaking"[All Fields]) AND ("developing countries"[MeSH Terms] OR ("developing"[All Fields] AND "countries"[All Fields]) OR "developing countries"[All Fields] OR ("low"[All Fields] AND "middle"[All Fields] AND "income"[All Fields] AND "countries"[All Fields]) OR "low and middle income countries"[All Fields])<br><br>**Translations**<br><br>**Barriers:** "barrier"[All Fields] OR "barrier's"[All Fields] OR "barriers"[All Fields]<br>**evidence:** "evidence"[All Fields] OR "evidences"[All Fields] OR "evident"[All Fields] OR "evidently"[All Fields]<br>**uptake:** "uptake"[All Fields] OR "uptakes"[All Fields] OR "uptaking"[All Fields]<br>**health:** "health"[MeSH Terms] OR "health"[All Fields] OR "health's"[All Fields] OR "healthful"[All Fields] OR "healthfulness"[All Fields] OR "healths"[All Fields]<br>**policymaking:** "policymaker"[All Fields] OR "policymaker's"[All Fields] OR "policymakers"[All Fields] OR "policymaking"[All Fields]<br>**low- and middle-income countries:** "developing countries"[MeSH Terms] OR ("developing"[All Fields] AND "countries"[All Fields]) OR "developing countries"[All Fields] OR ("low"[All Fields] AND "middle"[All Fields] AND "income"[All Fields] AND "countries"[All Fields]) OR "low and middle income countries"[All Fields]<br><br>**Warnings**<br><br>Barriers **to** evidence uptake **for** health policymaking **in** low- and middle-income countries<br><br>**Stop words:** to, for, in		

Showing 1 to 15 of 15 entries

FOLLOW NCBI

Connect with NLM

National Library of Medicine
8600 Rockville Pike
Bethesda, MD 20894

Web Policies
FOIA
HHS Vulnerability Disclosure

Help
Accessibility
Careers

NLM NIH HHS USA.gov

Cochrane Library

Advanced Search

Search

Save search | View saved searches | ? Search help

Did you know you can now select fields from Search manager using the S button (next to the search box)? Search manager lets you add unlimited search lines, view results per line and access the MeSH browser using the new MeSH button.

Title Abstract Keyword | Barriers OR challenges OR "influencing factors" OR bottlenecks AND "evidence uptake" "research u

(Word variations have been searched)

+

Search limits

Send to search manager

Run search

Clear all

Filter your results

Cochrane Reviews 1638	Cochrane Protocols 34	Trials 76549	Editorials 96	Special Collections 3	Clinical Answ 29	More

1638 Cochrane Reviews matching **Barriers OR challenges OR "influencing factors" OR bottlenecks AND "evidence uptake" "research uptake" OR "research evidence use" OR "evidence use" OR "evidence to policy translation" OR "research for policymaking" OR "evidence -informed policy" OR "evidence-based practice""" AND "low and middle income countries" OR LMICs OR "developing countries" OR "sub-Saharan Africa" OR "sub-Saharan countries" OR "low resource setting" OR "resource limited countries" in Title Abstract Keyword - (Word variations have been searched)**

Cochrane Database of Systematic Reviews

Issue 3 of 12, March 2023

Deselect all (1638) Export selected citation(s) Show all previews

Order by Publish Date - New To Old Results per page 25

1

Consumers' and health providers' views and perceptions of partnering to improve health services design, delivery and evaluation: a co-produced qualitative evidence synthesis

Bronwen Merner, Lina Schonfeld, Ariane Virgona, Dianne Lowe, Louisa Walsh, Cheryl Wardrope, Lisa Graham-Wisener, Vicki Xafis, Cinzia Colombo, Nora Refahi, Paul Bryden, Renee Chmielewski, Fiona Martin, Nancy M Messino, Anne Mussared, Lorraine Smith, Susan Biggar, Marie Gill, David Menzies, Carolyn M Gaulden, Leia Earnshaw, Leslie Arnott, Naomi Poole, Rebecca E Ryan, Sophie Hill

Qualitative Review 14 March 2023 Open access

Show preview

2

Botulinum toxin for the treatment of strabismus

Angeles R. Bort-Martí, Fiona J Rowe, Laura Ruiz Sifre, Sueko M Ng, Sylvia Bort-Martí, Vicente Ruiz Garcia

Intervention Review 14 March 2023 New search Conclusions changed

Show PICOs **Show preview**

3

Medical interventions for traumatic hyphema

Fasika A Woreta, Kristina B Lindsley, Almutez Gharaibeh, Sueko M Ng, Roberta W Scherer, Morton F Goldberg

Intervention Review 13 March 2023 New search

Show PICOs **Show preview**

4

Planned hospital birth compared with planned home birth for pregnant women at low risk of complications

Ole Olsen, Jette A Clausen

Intervention Review 8 March 2023 New search Conclusions changed

Show PICOs **Show preview**

5

Interventions for improving adherence to iron chelation therapy in people with sickle cell disease or thalassaemia

Louise J Geneen, Carolyn Dorée, Lise J Estcourt

Intervention Review 6 March 2023 New search

Show PICOs **Show preview**

6

Ataluren and similar compounds (specific therapies for premature termination codon class I mutations) for cystic fibrosis

Aisha A Aslam, Ian P Sinha, Kevin W Southern

Intervention Review 3 March 2023 New search

Show PICOs **Show preview**

7 ☑

Intratympanic corticosteroids for Ménière's disease

Katie E Webster, Ambrose Lee, Kevin Galbraith, Natasha A Harrington-Benton, Owen Judd, Diego Kaski, Otto R Maarsingh, Samuel MacKeith, Jaydip Ray, Vincent A Van Vugt, Brian Westerberg, Martin J Burton

Intervention Review 27 February 2023 Open access

Show PICOs **Show preview**

8 ☑

Methods for informing people with amyotrophic lateral sclerosis/motor neuron disease of their diagnosis

Paolo Bongioanni, Gian Domenico Borasio, David J Oliver, Andrea Romagnoli, Karl P Kapitza, Katie Sidle, Francesco Tramonti

Intervention Review 22 February 2023

Show PICOs **Show preview**

9 ☑

Interventions for myopia control in children: a living systematic review and network meta-analysis

John G Lawrenson, Rakhee Shah, Byki Huntjens, Laura E Downie, Gianni Virgili, Rohit Dhakal, Pavan K Verkicharla, Dongfeng Li, Sonia Mavi, Ashleigh Kernohan, Tianjing Li, Jeffrey J Walline

Intervention Review 16 February 2023 Open access

Show PICOs **Show preview**

10 ☑

Antenatal dietary supplementation with myo-inositol for preventing gestational diabetes

Soana K Motuhifonua, Luling Lin, Jane Alsweiler, Tineke J Crawford, Caroline A Crowther

Intervention Review 15 February 2023 New search

Show PICOs **Show preview**

11 ☑

Factors that influence the provision of home-based rehabilitation services for people needing rehabilitation: a qualitative evidence synthesis

Marcela Velez, Luz Helena Lugo-Agudelo, Daniel F. Patiño Lugo, Claire Glenton, Ana M Posada, Luisa Fernanda Mesa Franco, Stefano Negrini, Carlotte Kiekens, Maria Alejandra Spir Brunal, Anne-Stine Bergquist Roberg, Kelly Mariana Cruz Sarmiento

Qualitative Review 10 February 2023 Open access
Show preview

12 ☑

Behavioural and cognitive-behavioural interventions for outwardly directed aggressive behaviour in people with intellectual disabilities

David Prior, Soe Win, Angela Hassiotis, Ian Hall, Michele A Martiello, Afia K Ali

Intervention Review 6 February 2023 New search
Show PICOs **Show preview**

13 ☑

Digital technology for monitoring adherence to inhaled therapies in people with cystic fibrosis

Sherie Smith, Rebecca Calthorpe, Sophie Herbert, Alan R Smyth

Intervention Review 3 February 2023
Show PICOs **Show preview**

14 ☑

Treatment of dental and orthodontic complications in thalassaemia

Priti Mulimani, Adinegara BL Abas, Laxminarayan Karanth, Raffaella Colombatti, Palna Kulkarni

Intervention Review 2 February 2023 New search
Show PICOs **Show preview**

15 ☑

Convalescent plasma for people with COVID-19: a living systematic review

Claire Iannizzi, Khai Li Chai, Vanessa Piechotta, Sarah J Valk, Catherine Kimber, Ina Monsef, Erica M Wood, Abigail A Lamikanra, David J Roberts, Zoe McQuilten, Cynthia So-Osman, Aikaj Jindal, Nora Cryns, Lise J Estcourt, Nina Kreuzberger, Nicole Skoetz

Intervention Review 1 February 2023 New search Conclusions changed
Show PICOs **Show preview**

16 ☑

Cognitive stimulation to improve cognitive functioning in people with dementia

Bob Woods, Harleen Kaur Rai, Emma Elliott, Elisa Aguirre, Martin Orrell, Aimee Spector

Intervention Review 31 January 2023 New search Conclusions changed
Show PICOs **Show preview**

17 ☑

Hyperimmune immunoglobulin for people with COVID-19

Catherine Kimber, Sarah J Valk, Khai Li Chai, Vanessa Piechotta, Claire Iannizzi, Ina Monsef, Erica M Wood, Abigail A Lamikanra, David J Roberts, Zoe McQuilten, Cynthia So-Osman, Lise J Estcourt, Nicole Skoetz

Intervention Review 26 January 2023
Show PICOs **Show preview**

18 ☑

Interventions to improve sanitation for preventing diarrhoea

Valerie Bauza, Wenlu Ye, Jiawen Liao, Fiona Majorin, Thomas Clasen

Intervention Review 25 January 2023 Open access
Show PICOs **Show preview**

19 ☑

Remdesivir for the treatment of COVID-19

Felicitas Grundeis, Kelly Ansems, Karolina Dahms, Volker Thieme, Maria-Inti Metzendorf, Nicole Skoetz, Carina Benstoem, Agata Mikolajewska, Mirko Griesel, Falk Fichtner, Miriam Stegemann

Intervention Review 25 January 2023 New search Conclusions changed
Show PICOs **Show preview**

20 ☑

Corticosteroids for the management of cancer-related fatigue in adults with advanced cancer

Amy Sandford, Alison Haywood, Kirsty Rickett, Phillip Good, Sohil Khan, Karyn Foster, Janet R Hardy

Intervention Review 23 January 2023
Show PICOs **Show preview**

21 ☑

Corticosteroid implants for chronic non-infectious uveitis

Amit Reddy, Su-Hsun Liu, Christopher J Brady, Pamela C Sieving, Alan G Palestine

Intervention Review 16 January 2023 New search Conclusions changed
Show PICOs **Show preview**

22 ☑

Systemic opioid regimens for postoperative pain in neonates

Mari Kinoshita, Israel Junior Borges do Nascimento, Lea Styrmisdóttir, Matteo Bruschettini

Intervention Review 16 January 2023
Show PICOs **Show preview**

23 ☑

Telerehabilitation for people with low vision

Ava K Bittner, Patrick D Yoshinaga, Thanitsara Rittiphairoj, Tianjing Li

Intervention Review 13 January 2023 New search Conclusions changed
Show PICOs **Show preview**

24

Vitamin D as an adjunct to antibiotics for the treatment of acute childhood pneumonia

Rashmi R Das, Meenu Singh, Sushree S Naik

Intervention Review 12 January 2023 New search

Show PICOs **Show preview**

25

Psychological interventions for women with non-metastatic breast cancer

Ghufran A Jassim, Sally Doherty, David L Whitford, Ali S Khashan

Intervention Review 11 January 2023 New search

Show PICOs **Show preview**

1 2 3 4 5 6 7 8 9 10 11 Next

Search History/Alerts

Print Search History Retrieve Searches Retrieve Alerts Save Searches / Alerts

Select / deselect all | Search with AND | Search with OR | Delete Searches

Refresh Search Results

Search ID#	Search Terms	Search Options	Actions
S2	TX (Barriers OR challenges OR "influencing factors" OR bottlenecks) AND TX (("evidence uptake" OR "research uptake" OR "research evidence use" OR "evidence use" OR "evidence to policy translation" OR "research for policymaking" OR "evidence -informed policy" OR "evidence-based practice") AND TX ("low and middle income countries" OR LMICs OR "developing countries" OR "sub-Saharan Africa" OR "sub-Saharan countries" OR "low resource setting" OR "resource limited countries")	**Expanders** - Apply equivalent subjects **Narrow by SubjectMajor:** - health policy **Search modes** - Boolean/Phrase	View Results (29) View Details Edit
S1	TX (Barriers OR challenges OR "influencing factors" OR bottlenecks) AND TX (("evidence uptake" OR "research uptake" OR "research evidence use" OR "evidence use" OR "evidence to policy translation" OR "research for policymaking" OR "evidence -informed policy" OR "evidence-based practice") AND TX ("low and middle income countries" OR LMICs OR "developing countries" OR "sub-Saharan Africa" OR "sub-Saharan countries" OR "low resource setting" OR "resource limited countries")	**Expanders** - Apply related words; Also search within the full text of the articles; Apply equivalent subjects **Search modes** - Boolean/Phrase	View Results (604) View Details Edit

1. Community health workers and health equity in **low-** and **middle-inc**[illegible]**ne countries**: systematic review and recommendations for **policy** and **practice**.

   (includes abstract) Ahmed, Sonia; Chase, Liana E.; Wagnild, Janelle; Akhter, Nasima; Sturridge, Scarlett; Clarke, Andrew; Chowdhary, Pari; Mukami, Diana; Kasim, Adetayo; Hampshire, Kate International Journal for Equity in Health, 11/12/2022; 21(1): 1-30. 30p. (Article - **research**, systematic review, tables/charts) ISSN: 1475-9276

   Subjects: Community Health Workers; Health Inequities; **Low** and **Middle Income Countries**; Health Services Accessibility; World Health; **Health Policy**

   Get it @ King's

2. Implementation outcomes of **policy** and programme innovations **to** [illegible]vent obstetric haemorrhage in **low-** and **middle-income countries**: a systematic review.

   (includes abstract) Ryan, Nessa; Vieira, Dorice; Goffman, Dena; Bloch, Evan M; Akaba, Godwin O; D'mello, Brenda S; Egekeze, Chioma; Snyder, Anya; Lyimo, Magdalena; Nnodu, Obiageli; Peprah, Emmanuel **Health Policy** & Planning, Nov2020; 35(9): 1208-1227. 20p. (journal article - **research**, systematic review, tables/charts) ISSN: 0268-1080 PMID: NLM32995854

   Subjects: Developing Countries; **Health Policy** Economics; Preventive Health Care Statistics and Numerical Data; Postpartum Hemorrhage Prevention and Control; Preventive Health Care Economics; Female

   Get it @ King's

3. **Policy** limitations and constraints **to** exclusive breastfeeding among working mothers in **low-** and **middle-income countries**: the Nigeria case.

   (includes abstract) Nwaodu-Ufomba, Linda African Journal of Midwifery & Women's Health, 2022; 16(2): 1-10. 10p. (Article - **research**, systematic review, tables/charts) ISSN: 1759-7374

   Subjects: Breast Feeding Nigeria; Mothers, Working; **Low** and **Middle Income Countries**; **Health Policy**

   Linked Full Text

4. **Research** priorities **to** support **evidence-informed** policies and adv[illegible]cy for access **to** safe abortion care in **sub-Saharan Africa**.

(includes abstract) Ajayi, Anthony Idowu; Ouedraogo, Ramatou; Juma, Kenneth; Kibunja, Grace; Cheruiyot, Collins; Mwoka, Meggie; Igonya, Emmy Kageha; Opondo, Winnie; Otukpa, Emmanuel; Kabiru, Caroline W.; Ushie, Boniface Ayanbekongshie Sexual & Reproductive Health Matters, Dec 2021; 29(1): 5-8. 4p. (Article) ISSN: 2641-0397

Subjects: Abortion, Induced Legislation and Jurisprudence; **Health Policy**; **Policy** Making; **Research** Priorities; Medical **Practice**, **Research-Based**; Health Services Accessibility

Get it @ King's

5. Behavior analytic interventions for children with autism: **Policy** and **practice** in the United Kingdom and China.

(includes abstract) Liao, Yini; Dillenburger, Karola; Hu, Xiaoyi Autism: The International Journal of **Research** & **Practice**, Jan2022; 26(1): 101-120. 20p. (Article - **research**, tables/charts) ISSN: 1362-3613

Subjects: Autistic Disorder In Infancy and Childhood; Behavior Therapy; Health Personnel Psychosocial **Factors**; Parents Psychosocial **Factors**; **Health Policy** United Kingdom; Applied Behavior Analysis; **Health Policy** China; Child: 6-12 years

Get it @ King's

6. EVITA 2.0, an updated framework for understanding **evidence-base** mental **health policy** agenda-**setting**: tested and **informed** by key informant interviews in a multilevel comparative case study.

(includes abstract) Votruba, Nicole; Grant, Jonathan; Thornicroft, Graham Health **Research Policy** & Systems, 3/10/2021; 19(1): 1-15. 15p. (journal article) ISSN: 1478-4505 PMID: NLM33691696

Subjects: Policy Making; **Health Policy**

Get it @ King's

7. Institutional capacity **to** generate and **use evidence** in **LMICs**: curre state and opportunities for HPSR.

(includes abstract) Shroff, Zubin Cyrus; Javadi, Dena; Gilson, Lucy; Kang, Rockie; Ghaffar, Abdul Health **Research Policy** & Systems, 11/9/2017; 15 1-11. 11p. (journal article - **research**, tables/charts) ISSN: 1478-4505 PMID: NLM29121958

Subjects: Organizational Development Administration; **Health Policy**; **Policy** Making; **Developing Countries**; Professional **Practice**, **Evidence-Based** Administration

Get it @ King's

8. **Barriers** and Facilitators **to** the Successful Transition of Adolescents ving with HIV from Pediatric **to** Adult Care in **Low** and **Middle-Income Countries**: A Systematic Review and **Policy** Analysis.

(includes abstract) Jones, Cameron; Ritchwood, Tiarney D.; Taggart, Tamara AIDS & Behavior, Sep2019; 23(9): 2498-2513. 16p. (Article - **research**, systematic review, tables/charts) ISSN: 1090-7165

Subjects: HIV-Positive Persons In Adolescence; Transitional Care; **Low** and **Middle Income Countries**; Health Services Accessibility; **Health Policy**; Adolescent: 13-18 years

Get it @ King's

9. How do decision-makers **use evidence** in community **health policy** d financing decisions? A qualitative study and conceptual framework in four African **countries**.

(includes abstract) Kumar, Meghan Bruce; Taegtmeyer, Miriam; Madan, Jason; Ndima, Sozinho; Chikaphupha, Kingsley; Kea, Aschenaki; Barasa, Edwine **Health Policy** & Planning, Aug2020; 35(7): 799-809. 11p. (journal article - **research**, tables/charts) ISSN: 0268-1080 PMID: NLM32516361

Subjects: **Health Policy** Economics; Public Health Methods

Get it @ King's

10. The role of health system governance in strengthening the rural heal insurance system in China.

(includes abstract) Beibei Yuan; Weiyan Jian; Li He; Bingyu Wang; Balabanova, Dina International Journal for Equity in Health, 5/23/2017; 16 1-20. 20p. (Article - **research**, systematic review, tables/charts) ISSN: 1475-9276

Subjects: Health Services Administration China; Rural Health Services; Insurance, Health History; **Health Policy** Evaluation

Get it @ King's

11. Public-Private Partnership **Policy** in Primary Health Care: A Scoping Review.

(includes abstract) Tabrizi, Jafar Sadegh; Azami-aghdash, Saber; Gharaee, Hojatolah Journal of Primary Care & Community Health, 8/25/2020; 11 1-17. 17p. (Article - pictorial, **research**, systematic review, tables/charts) ISSN: 2150-1319

Subjects: Primary Health Care; Public Sector; Private Sector; Collaboration; **Health Policy**

Get it @ King's

12. The moral and social narratives of sexual and reproductive health in Kenya: a case of adolescents and young people pre- and within the MDG era.

(includes abstract) Akwara, Elsie; Idele, Priscilla Reproductive Health, 5/26/2020; 17(1): 1-12. 12p. (Article - **research**, systematic review, tables/charts) ISSN: 1742-4755

Subjects: Morals Kenya; Social Behavior Kenya; **Health Policy** Kenya; Sexual Health Legislation and Jurisprudence; Reproductive Health Legislation and Jurisprudence; Adolescent: 13-18 years

Times Cited in this Database: (1)

Get it @ King's

13. **Policy** dialogues: facilitators' perceived role and influence.

(includes abstract) Biermann, Olivia; Kuchenmüller, Tanja; Panisset, Ulysses; Leys, Mark International Journal of Health Governance, 2018; 23(2): 120-133. 14p. (Article - **research**, tables/charts) ISSN: 2059-4631

Subjects: Policy Making Labor Supply; **Health Policy**; Professional **Practice**, **Evidence-Based**; Professional Role; Leadership; Female; Male

Linked Full Text

14. Cervical cancer screening decentralized **policy** adaptation: an Africa rural-context-specific systematic literature review.

(includes abstract) Rahman, R.; Clark, M. D.; Collins, Z.; Traore, F.; Dioukhane, E. M.; Thiam, H.; Ndiaye, Y.; De Jesus, E. L.; Danfakha, N.; Peters, K. E.; Komarek, T.; Linn, A. M.; Linn, P. E.; Wallner, K. E.; Charles, M.; Hasnain, M.; Peterson, C. E.; Dykens, J. A. Global Health Action, 2019; 12(1): 1-11. 11p. (Article - **research**, systematic review, tables/charts) ISSN: 1654-9716

Subjects: Cervix Neoplasms Diagnosis; Cancer Screening Methods; **Health Policy Africa**; Quality Improvement; Program Implementation

Get it @ King's

15. Public-Private Partnership **Policy** in Primary Health Care: A Scoping Review.

(includes abstract) Tabrizi, Jafar Sadegh; Azami-aghdash, Saber; Gharaee, Hojatolah Journal of Primary Care & Community Health, Jan-Dec2020; 1-17. 17p. (Article - pictorial, **research**, systematic review, tables/charts) ISSN: 2150-1319

Subjects: Primary Health Care; Public Sector; Private Sector; Collaboration; **Health Policy**; Program Evaluation

Get it @ King's

16. **Challenges** in **policy** reforms for non-communicable diseases: the c[illegible]e of diabetes in Kenya.

(includes abstract) Shiroya, Veronica; Neuhann, Florian; Müller, Olaf; Deckert, Andreas Global Health Action, 2019; 12(1): 1-11. 11p. (Article - **research**, systematic review, tables/charts) ISSN: 1654-9716

Subjects: Health Policy; Noncommunicable Diseases; Diabetes Mellitus Prevention and Control

Get it @ King's

17. Tackling bad air and promoting prevention and care for chronic lung diseases in **sub-Saharan Africa**...14th European Public Health Conference (Virtual), Public health futures in a changing world, November 10-12, 2021.

(includes abstract) Ku, G. M. V.; Da Silveira, V. C.; Kegels, G.; Develtere, P.; Nemery, B.; Doussou, J. P.; Bossyns, P.; Gyselinck, K. European Journal of Public Health, 2021 Supplement; 31 iii409-iii409. 1/3p. (Article - abstract, proceedings, **research**, systematic review) ISSN: 1101-1262

Subjects: Air Pollution, Indoor Prevention and Control; Chronic Disease; Lung Diseases Prevention and Control; Patient Care; Social Determinants of Health; Health Care Delivery; Health Promotion; **Health Policy Africa** South of the Sahara

Get it @ King's

18. Ageing as a global public health **challenge**: From complexity reducti[illegible] **to** aid effectiveness.

Esser, Daniel E.; Ward, Patricia S. Global Public Health, Aug2013; 8(7): 745-768. 24p. (Journal Article - meta analysis, **research**, tables/charts) ISSN: 1744-1692 PMID: NLM23914730

Subjects: Aging; **Health Policy**; Humanitarian Aid **Developing Countries**; Public Health; Age Specific Care Economics

Linked Full Text

19. **Developing** the National Knowledge Platform in India: a **policy** and institutional analysis.

(includes abstract) Sriram, Veena; Bennett, Sara; Raman, V. R.; Sheikh, Kabir Health **Research Policy** & Systems, 2/20/2018; 16 1-1. 1p. (journal article) ISSN: 1478-4505 PMID: NLM29463256

Subjects: Public Health; **Research**, Medical; Medical **Practice**, **Evidence-Based**; Cooperative Behavior; Knowledge; Health Care Delivery; **Health Policy**

Get it @ King's

20. A roadmap for strengthening **evidence-informed health policy**-mal[illegible]g in Iran: protocol for a **research** programme.

(includes abstract) Sajadi, Haniye Sadat; Majdzadeh, Reza; Yazdizadeh, Bahareh; Mohtasham, Farideh; Mohseni, Mahsa; Doshmangir, Leila; Lavis, John Health **Research Policy** & Systems, 5/17/2019; 17(1): N.PAG-N.PAG. 1p. (journal article) ISSN: 1478-4505 PMID: NLM31101047

Subjects: Developing Countries; Government Programs; **Research**, Medical; **Health Policy**; Health Care Delivery Standards; Professional **Practice**, **Evidence-Based** Standards; **Policy** Making

Get it @ King's

EBSCO Connect | Privacy Policy | A/B Testing | Terms of Use | Copyright | Cookie Policy | Manage my Cookies

powered by EBSCO*host*

© 2023 EBSCO Industries, Inc. All rights reserved.

TRY THE NEW OVID INTERFACE

Ovid®

Wolters Kluwer

My Account Support & Training Ovid® Search Builder Help Feedback Logoff

Search Journals Books My Workspace Multimedia

▼ **Search History** (23)

View Saved

	# ▲	Searches	Results	Type	Actions	Annotations
	1	Barriers.mp. [mp=abstract, title, original title, heading words, cabicodes words]	42298	Advanced	Display Results | More	
	2	challenges.mp. [mp=abstract, title, original title, heading words, cabicodes words]	74474	Advanced	Display Results | More	
	3	"influencing factors".mp. [mp=abstract, title, original title, heading words, cabicodes words]	7216	Advanced	Display Results | More	
	4	bottlenecks.mp. [mp=abstract, title, original title, heading words, cabicodes words]	1206	Advanced	Display Results | More	
	5	"evidence uptake".mp. [mp=abstract, title, original title, heading words, cabicodes words]	30	Advanced	Display Results | More	
	6	"research uptake".mp. [mp=abstract, title, original title, heading words, cabicodes words]	30	Advanced	Display Results | More	
	7	1 or 2 or 3	118437	Advanced	Display Results | More	
	8	"research evidence use".mp. [mp=abstract, title, original title, heading words, cabicodes words]	5	Advanced	Display Results | More	
	9	"evidence use".mp. [mp=abstract, title, original title, heading words, cabicodes words]	82	Advanced	Display Results | More	
	10	"evidence to policy translation".mp. [mp=abstract, title, original title, heading words, cabicodes words]	1	Advanced	Display Results | More	

Contract

☐	11	"research for policymaking".mp. [mp=abstract, title, original title, heading words, cabicodes words]	33	Advanced	Display Results | More	
☐	12	"evidence-informed policy".mp. [mp=abstract, title, original title, heading words, cabicodes words]	140	Advanced	Display Results | More	
☐	13	"evidence-based practice".mp. [mp=abstract, title, original title, heading words, cabicodes words]	1109	Advanced	Display Results | More	
☐	14	8 or 9 or 10 or 11 or 12 or 13	1346	Advanced	Display Results | More	
☐	15	7 and 14	313	Advanced	Display Results | More	
☐	16	"low and middle income countries".mp. [mp=abstract, title, original title, heading words, cabicodes words]	12482	Advanced	Display Results | More	
☐	17	"developing countries".mp. [mp=abstract, title, original title, heading words, cabicodes words]	48297	Advanced	Display Results | More	
☐	18	"sub-Saharan Africa".mp. [mp=abstract, title, original title, heading words, cabicodes words]	18947	Advanced	Display Results | More	
☐	19	"sub-Saharan countries".mp. [mp=abstract, title, original title, heading words, cabicodes words]	500	Advanced	Display Results | More	
☐	20	"low resource setting".mp. [mp=abstract, title, original title, heading words, cabicodes words]	541	Advanced	Display Results | More	
☐	21	"resource limited countries".mp. [mp=abstract, title, original title, heading words, cabicodes words]	740	Advanced	Display Results | More	
☐	22	16 or 17 or 18 or 19 or 20 or 21	76727	Advanced	Display Results | More	
☐	23	15 and 22	29	Advanced	Display Results | More	

Save Remove

Combine with: AND

OR

Save All | Edit | Create RSS | Create Auto-Alert | View Saved

Email All Search History | Copy Search History Link | Copy Search History Details

Basic Search | **Advanced Search** | Find Citation | Search Tools | Search Fields | Multi-Field Search

1 Resource selected | Hide | Change

Global Health 1973 to 2023 Week 11

Keyword Author Title Journal

Enter keyword or phrase
(* or $ for truncation)

Search

Include Multimedia

Map Term to Subject Heading

Limits *(expand)*

To search Open Access content on Ovid, go to Basic Search.

Options

Search Information

You searched:

15 and 22

Search terms used:

barriers
challenges
developing
countries
evidence
to
policy
translation
use
evidence-based
practice
evidence-informed
influencing
factors
low
and
middle
income
resource
setting
research
for
policymaking
limited
sub-saharan
africa

Search Returned:

29 text results

Sort By:

-

Customize Display

Filter By

Add to Search History

Selected Only (29)

▼ Years

All Years

Current year

Past 3 years

Past 5 years

▶ Specific Year Range

▶ Subject
▶ Author
▶ Journal
▶ Book
▶ Publication Type

My Projects

+ New Project

No projects available.

Print Email Export + My Projects Keep Selected

All Range

Clear

10 Per Page 1 Go

Next ›

1.
Cocreation in health workforce planning to shape the future of the health care system in the Philippines.

Liwanag, H. J.; Uy, J.; Politico, M. R.; Padilla, M. J.; Arzobal, M. C.; Manuel, K.; Cagouia, A. L.; Tolentino, P.; Frahsa, A.; Ronquillo, K.

Global Health: Science and Practice; 2022. 10(6). 47 ref.

[Journal article]

Abstract
Cite + My Projects
+ Annotate
Annotation(s)

Abstract Reference
Complete Reference

Find Similar
Find Citing Articles

Library Holdings
Get it @ King's

2.
Health policy and systems research capacities in Ethiopia and Ghana: findings from a self-assessment.

Ta[illegible]
Witthayapipopsakul, W.; Panichkriangkrai, W.

Global Health: Science and Practice; 2022. 10(Suppl. 1). 50 ref.

[Journal article]

Abstract
Cite + My Projects

+ Annotate

Annotation(s)

Abstract Reference
Complete Reference

Find Similar
Find Citing Articles

Library Holdings
Get it @ King's

3.
A qualitative study of evidence-based therapeutic process in mental health services in Ghana- context-mechanisms-outcomes.

Badu, E.; O'Brien, A. P.; Mitchell, R.; Osei, A.

BMC Health Services Research; 2021. 21(1013):(25 September 2021). 47 ref.

[Journal article]

Abstract
Cite + My Projects
+ Annotate

Annotation(s)

Abstract Reference
Complete Reference

Find Similar
Find Citing Articles

Library Holdings
Get it @ King's

4.
Barriers to evidence-based practice implementation in physiotherapy: a systematic review and meta-analysis.

Paci, M.; Faedda, G.; Ugolini, A.; Pellicciari, L.

International Journal for Quality in Health Care; 2021. 33(2). 72 ref.

[Journal article]

Abstract
Cite + My Projects
+ Annotate

Annotation(s)

Abstract Reference
Complete Reference

Find Similar
Find Citing Articles

Library Holdings
Get it @ King's

5.
Human resources for health-related challenges to ensuring quality newborn care in low- and middle-income countries: a scoping review.

Bolan, N.; Cowgill, K. D.; Walker, K.; Kak, L.; Shaver, T.; Moxon, S.; Lincetto, O.

Global Health: Science and Practice; 2021. 9(1):160-176. 90 ref.

[Journal article]

Abstract
Cite + My Projects
+ Annotate
Annotation(s)

Abstract Reference
Complete Reference

Find Similar
Find Citing Articles

Library Holdings
Get it @ King's

6.
Developing and implementing the family nurse practitioner role in Eswatini: implications for education, practice, and policy.

Dlamini, C. P.; Khumalo, T.; Nkwanyana, N.; Mathunjwa-Dlamini, T. R.; Macera, L.; Nsibandze, B. S.; Kaplan, L.; Stuart-Shor, E. M.

Annals of Global Health; 2020. 86(1). 38 ref.

[Journal article]

Abstract
Cite + My Projects
+ Annotate
Annotation(s)

Abstract Reference
Complete Reference

Find Similar
Find Citing Articles

Library Holdings
Get it @ King's

7.
Evidence-based practice and rural health service delivery: knowledge and barriers to adoption among clinical nurses in Ghana.

Yiridomoh, G. Y.; Dayour, F.; Bonye, S. Z.

Rural Society; 2020. 29(2):134-149. 43 ref.

[Journal article]

Abstract
Cite + My Projects
+ Annotate
Annotation(s)

Abstract Reference
Complete Reference

Find Similar
Find Citing Articles

Library Holdings
Get it @ King's

8.

Evidence-based practice and associated factors among health care providers working in public hospitals in Northwest Ethiopia during 2017.

Getenet Dessie; Dube Jara; Girma Alem; Henok Mulugeta; Tesfu Zewdu; Fasil Wagnew; Bigley, R.; Burrowes, S.

Current Therapeutic Research; 2020. 93. 45 ref.

[Journal article]

Abstract
Cite + My Projects
+ Annotate
Annotation(s)

Abstract Reference
Complete Reference

Find Similar
Find Citing Articles

Full Text
Get it @ King's

9.

Stroke rehabilitation in low-income and middle-income countries: a call to action.

Bernhardt, J.; Urimubenshi, G.; Gandhi, D. B. C.; Eng, J. J.

Lancet (British edition); 2020. 396(10260):1452-1462.

[Journal article]

Abstract
Cite + My Projects
+ Annotate
Annotation(s)

Abstract Reference
Complete Reference

Find Similar
Find Citing Articles

Library Holdings
Get it @ King's

10.

How do decision-makers use evidence in community health policy and financing decisions? A qualitative study and conceptual framework in four African countries.

Kumar, M. B.; Taegtmeyer, M.; Madan, J.; Ndima, S.; Chikaphupha, K.; Aschenaki Kea; Barasa, E.

Health Policy and Planning; 2020. 35(7):799-809. many ref.

[Journal article]

Abstract
Cite + My Projects
+ Annotate
Annotation(s)

Abstract Reference
Complete Reference

Find Similar
Find Citing Articles

Full Text
Get it @ King's

All Range

Clear

10 Per Page 1 Go

Next ›

Print Email Export + My Projects Keep Selected

English Français Italiano Deutsch 日本語 繁體中文 Español 简体中文 한국어

About Us Contact Us Privacy Policy Terms of Use

© 2023 Ovid Technologies, Inc. All rights reserved.

OvidUI_04.24.01.001, SourceID 679adf16c0e3bb0144991a540aba87f81c038fc2
