# Supplementary material for: Challenges for research uptake for health policymaking and practice in low- and middle-income countries: a scoping review
Source: Health Res Policy Syst. 2023 Dec 6;21:131. doi: 10.1186/s12961-023-01084-5 (PMC10699029; doi:10.1186/s12961-023-01084-5)
Supplement: Supplementary file 3 — Additional file 3. Data charting. [file 12961_2023_1084_MOESM3_ESM.docx]

Additional file 3: **Data charting**

description of relevant evidence included in scoping review of research uptake for health policymaking and practice in LMICs

| **Author, year** | **Aim** | **Study type** | **Key findings** | **Recommendation** |
| --- | --- | --- | --- | --- |
| Aaserud et al.,2005 | to examine the factors that might affect the translation of (RCT findings into policies and practice in LMICs | implementation research | inadequate and poorly implemented clinical guidelines; and lack of political support for policy change. | This makes it difficult to envisage any single intervention strategy that might be used to promote the uptake of research findings |
| Abekah-Nkrumah et al., 2018 | to examine the production, dissemination and utilization of reproductive and child health-related evidence to inform policy formulation in Ghana’s health sector. | Review evidence translation in Ghana | absence of a robust institutional-wide mechanism for collating research needs and communicating these to researchers, communicating research findings in forms that are friendlier to policy-makers and the inability to incorporate funding for research into the budget of the health sector. | admonishing the Ministry of Health and its agencies to leverage on the strengthening of existing opportunities to improve research uptake |
| Abubakar et al., 2021 | To present co-production of evidence between political decision-makers, health policymakers and academics | Rapid review: lesson from covid | limited transparency, bureaucratic obstacles, larger systemic obstacles included a complex multitiered health system, fragmented decision-making structures and limited funding for implementation. | The co-production of evidence examining the broader public health impact, with synthesis by multidisciplinary teams, is essential to meeting the social and public health challenges |
| Abu‑Odah et al., 2022 | provides a platform for providing PC as part of the Palestinian healthcare system by exploring policymakers’ perspectives on PC, an essential step to developing a PC programme. | Qualitative implementation research | the development goals are not clearly defined, and the plan’s capabilities are inadequate. Several challenges to the provision of PC were found to relate to issues of education and training, the allocation of funding, and the availability of medications. | Developing policies aligned with national laws could help enhance health services to patients and their families and resolve several challenges. Cooperating with national and international institutions in seeking funding. |
| Afshari et al., 2020 | aimed to draw lessons for strengthening linkages with a wide range of actors and stakeholders | Systematic review | global collective action, key actors, their interests in the problem, potential negotiation process, and potential scenarios for collective action were identified, and categorized into three groups (1) core diplomacy, (2) multi-stakeholder diplomacy, and (3) informal diplomacy | Development and adoption of a global policy to tackle the rise in NCDs in developed and developing countries require policymakers and political leaders that participate in GHD |
| Ager & Zarowsky, 2015 | To assess the health research capacity strengthening program of work across sub-Saharan Africa. | Systematic review | identify tensions between efforts to embrace the global ‘Community of Science’ and the promotion and protection of national and institutional agendas in an unequal global health research environment. | A nuanced understanding of the dynamics and implications of the uneven global health research landscape is required, along with a willingness to explore pragmatic models that seek to balance these competing drivers |
| Akhlaq et al., 2016 | to understand the barriers and facilitators to the implementation and adoption of HIE in LMICs | Systematic review | lack of importance given to data in decision-making, corruption and insecurity, lack of training and poor infrastructure was considered to be major challenges to implementing HIE, but strong leadership and clear policy direction coupled with the financial support to acquire essential technology, improve the communication network, and provide training for staff all helped to promote implementation | The body of work also high-lighted how implementers of HIE needed to take into account local needs to ensure that stake-holders saw HIE as relevant and advantageous |
| Al-Bader et al., 2010 | To collect results of this study to be of interest to a wide range of stakeholders | Qualitative implementation research | the practical challenges for innovators on the ground, and suggest potentially helpful policies, funding streams, and other support systems. For African nations, health innovation represents an opportunity to increase domestic capacity to solve health challenges; for international funders, it is an opportunity to move beyond foreign aid and dependency. | link entrepreneurs, scientists, funders, and policy-makers into a network to share opportunities and challenges; and ultimately to better support and stimulate African-led health innovation |
| Ali et al., 2021 | describe the educational approach and processes used, and discuss successes challenges, and lessons learned | Cross-sectional implementation research | Most students reported benefits from the rural internship experience but were challenged by the isolation of the rural site, and felt unsupported by their supervisors, undermining students’ experiences and potentially the quality of the research. Financial barriers were also reported as challenges by students, even among those who received bursaries. | The partnership was successful in establishing a Master Programme in Nutritional Epidemiology increasing the number of nutrition researchers in the DRC. This approach could be used in other LMIC settings to address health and nutrition challenges. |
| Aranda-Jan et al. 2014 | review documents strengths, weaknesses, opportunities, and threats (SWOT) of mHealth projects in Africa. | Systematic review | mHealth projects demonstrate positive health-related outcomes and their success is based on the accessibility, acceptance and low-cost of the technology, effective adaptation to local contexts, strong stakeholder collaboration, and government involvement | innovative approach to delivering health services, fast-growing technology, research opportunities for scale-up, evaluating cost-effectiveness and impacts on the overall health system. |
| Baatiema et al., 2017a | examined the overall barriers and enablers, as perceived by health professionals which affect how evidence-based practice guidelines | Systematic review | Poor organizational or institutional level support, health professionals’ limited skills or competence to use a particular therapy, low level of awareness, familiarity or confidence in the effectiveness of a particular evidence-based therapy, limited medical facilities to support evidence uptake, inadequate peer support among health professionals’, complex nature of some stroke care therapies or guidelines and patient level barriers. | future interventions and health policy directions should be informed by these findings in order to optimize uptake of best practice acute stroke care. Further studies from low- to middle-income countries are needed to understand the barriers and enablers in such settings. |
| Baatiema et al., 2017b | to understand stroke care professionals’ views on the barriers which hinder the provision of optimal acute stroke care in Ghanaian hospital settings | Qualitative implementation research | barriers at the patient (financial constraints, delays, sociocultural or religious practices, discharge against medical advice, denial of stroke), health system (inadequate medical facilities, lack of stroke care protocol, limited staff numbers, inadequate staff development opportunities), health professionals (poor collaboration, limited knowledge of stroke care interventions) and broader national health policy (lack of political will) levels. | Barriers from low/middle-income countries differ substantially from those in high-income countries. For evidence-based acute stroke care in LMICs such as Ghana, health policy-makers and hospital managers need to consider the contrasts and uniqueness in these barriers in designing quality improvement interventions |
| Badu et al., 2021 | describes the processes involved in the delivery of best practice in Ghana. | Qualitative implementation research | evidence-based mental health services need establish therapeutic relationship building ensures effective interaction. | Government stakeholders and policymakers should prioritise policies, periodic monitoring and adequate financial incentives to support the mechanisms that promote technical competence and the building of therapeutic relationship. |
| Bennett et al., 2013 | Explore impact of FIC training on policy and practice, | Qualtative implementation research | Facilitators for this influence included the strong technical skills and scientific reputations of the trainees, and professional networks spanning research and policy communities. Barriers included the fact that trainees typically had not received training in research communication, relatively few policy makers had received scientific training, and institutional constraints that undermined alignment of research with policy needs. | strengthening research skills of scientists and developing strong in-country networks. Similar initiatives could be stimulated by investing more in the training of policy-makers, seeking to better align research with policy needs through more grants that are awarded directly to developing country institutions, addressing structural constraints, |
| Beran et al., 2015 | to identify the factors that contributed to informing and influencing policymakers with regards to this work. | Rapid review | close collaboration between the international team and local partners were strengths. Trust and a relationship with local partners were also seen as assets. All stakeholders valued the results and the credibility of the data generated. Local partners felt that more could have been done for dissemination. | The uptake of results was due to the credibility of the research which was influenced by a mix of the people involved, past assessments, trusted local partners, and the use of the results by knowledge brokers |
| Bigdeli et al., 2013 | to identify health system barriers, both at policy and implementation levels, to access and use | Mixed method | Barriers to access to and effective use of novel intervention occur at health facility level challenges due to lack of the medicine and reluctance of the health staff was reluctant to use it. | multi-stakeholder round-table meeting and an action plan for increasing access to this life-saving medicine was identified. |
| Biza et al., 2015 | explores the factors influencing provider uptake of the recommended package of ANC interventions in Mozambique | Qualitative study | The ANC package in Mozambique is not being fully implemented in the three study facilities, and a major barrier is poor functioning of the supply chain system. | ensuring evidence-based service that can be increasing the community understanding of the importance of care would improve demand for high quality services |
| Bulthuis et al., 2020 | to provide insights into the factors influencing the scale-up of public health interventions in LMICs. | Qualitative systematic literature review | factors influencing scale-up through changes in structure was the availability of financial, human and material resources. Inadequate supply chains were often barriers to scale-up. Advocacy activities positively influenced scale-up, and changes in the policy environment hindered or facilitated scale-up | availability of a strategic plan for scale-up, training and supervision, collaborations such as community participation and partnerships facilitated scale-up, as well as the availability of research and monitoring and evaluation data. |
| Cambe et al., 2022 | assessed barriers to the use of research evidence in the development and implementation of national health policies | Qualitative study | research generated does not always address questions that are relevant to the local health system development agenda. few of its publications support the translation of research evidence into policy. The research evidence generated is not locally relevant, | research institutions and policy makers in Mozambique collaborate on developing a platform that consolidates HPSR, making it more accessible and useful to policy makers. |
| Chan et al., 2015 | To explore relevant evidence for bridging the current division between research findings and their translation and uptake into policy and practice | Rapid review | the gap between research, policy, and practice is still far from acceptable, so much more research is required in this field, particularly as applied to the very big public health challenges that face LMICs | more collaborative research undertaken in partnership with practitioners, policymakers, and citizens will provide more learning that are amenable and feasible for wider implementation and uptake into policy and practice. |
| Chiu et al., 2019 | to understand the agencies’ initial difficulties with implementation and identify future challenges to help further policy development. | Qualitative study | Lack of policy integration between the government’s health and social departments; (2) Lack of understanding of the new policy among service agencies; (3) Lack of integration of vision and scope among service agencies | shed some light on the challenges to developing integrated LTC services and thus may help both policymakers and service providers find ways to overcome these challenges. |
| Cooper et al., 2015 | explores South African policymakers’ perspectives on public sector SRH-HIV policy integration, | Mixed method | integration challenges identified included a lack of policy and guidelines, inadequately trained providers, vertical programming, provider work overload, and a weak health system | The findings provide important insights for international, regional and national SRH-HIV policy and service integration initiatives. |
| Da Silva et al., 2019 | provides emerging researchers with an opportunity to describe their experiences of research opportunities. | Case study | Benefits included the receipt of research funding, hands-on training and mentorship, as well as exposure to networks and collaborative opportunities on a global scale | Governments and policymakers should prioritize educational policies to support the continuous development and international engagement of emerging researchers |
| Dalheim et al., 2012 | aimed to examine factors influencing the implementation of evidence-based practice | Cross-sectional study | The greatest barriers were lack of time and lack of skills to find and manage research evidence. Number of years since obtaining the last health professional degree influenced the use of sources of knowledge and self-reported barriers. | Skills in evidence-based practice seem to reduce barriers to using research evidence and to increase use of research evidence in clinical practice |
| Demment et al., 2015 | to highlight key findings, recommendations, and gaps in research and practice identified through a scoping study of recent reviews in breast and cervical cancer in LMICs. | Scoping review | a limited evidence base supports breast and cervical cancer control in LMICs. Governance, organizational improvement, workforces and community engagements are the strategy to improve evidence-based policy and practice | Further breast and cervical cancer prevention and control studies are necessary in LMICs. |
| Dizon et al., 2017 | describes barriers and enablers for allied health clinical practice guideline uptake in South African | Qualitative study | local barriers such as geography, AH training, workforce availability, scarce resources, an escalating number of patients requiring complex rehabilitation, and local knowledge. | Concerted attempts to implement locally relevant practice of primary care are required to improve widespread commitment to evidence-based care, as well as to plan efficient and effective service. |
| El-Jardali et al., 2014 | to gain a better understanding of the activities conducted by knowledge translation and policy (KTP) | Mixed method study | Deliberative dialogues informed by evidence briefs were identified as the most commendable tools by interviewees for enhancing EIHP. KTPs reported that they have contributed to increased awareness of the importance of EIHP and strengthened relationships among policymakers, stakeholders, retain human resources and secure funding. and researchers. Support from policymakers and international funders facilitated KTP activities, while the lack of skilled human resources to conduct EIHP activities impeded KTPs. | . Real-time lesson drawing from the experiences of KTPs can support improvements in the functioning of KTPs in the short term, while making the case for sustaining their work in the long term. Lessons learned can help to promote similar EIHP initiatives in other countries. KTPs hosted by universities highlighted the advantage of autonomy from political interests. |
| Erismann et al., 2021 | reports on five r4d research projects and shows how researchers engage with various stakeholders, including policy-makers, in order to assure uptake of the research results. | Qualitative study | different strategies that facilitate collaboration and communication between stakeholders, including policy-makers, and researchers. | it remains necessary to increase our understanding of the interests and motivations of the different actors involved in the process of influencing policy |
| Ezeanolue et al., 2018 | on findings from a structured group exercise conducted at the 2016 NISA Conference to identify (1) gaps in developing research capacity and (2) potential strategies to address these gaps. | Qualitative study | themes emerged for gaps (lack of sufficient funding, poor research focus in education, inadequate mentorship and training, inadequate research infrastructure, lack of collaboration between researchers, research-policy dissonance, lack of motivation for research, lack of leadership buy-in for research) and 7 themes emerged for strategies (increased funding for research, improved research education, improved mentorship and training, improved infrastructure for research, increased collaboration between academic/research institutions, greater engagement between researchers and policy-makers, greater leadership buy-in for research). | The gaps and strategies identified in this study represent pathways judged to be important in increasing research and implementation science capacity in Nigeria. The inclusion of perspectives and involvement of stakeholders who play different roles in policy, research and implementation activities makes these findings comprehensive, relevant and actionable, not only in Nigeria but in other similar LMICs |
| Feyissa et al., 2018 | to address concerns about the quality and standardisation of care received in primary care settings | Cross-sectional study | a research collaboration on health Systems Strengthening in sub-Saharan Africa) focused on health systems strengthening to support the scale-up of Ethiopian | Implementation and scale-up of the Ethiopian PHCG will be a key tool in primary healthcare transformation to achieve universal health coverage. |
| Goyet et al., 2014 | assessed the effectiveness of this KT intervention, with the goal of identifying the barriers to KT and suggest strategies to facilitate KT in similar settings. | Systematic review | involved stakeholders greatly differed and that there were several missed opportunities to translate on evidence into the adult pneumonia guideline. Seventeen facilitating factors and 18 potential barriers to KT were identified. Main barriers were related to the absence of a clear mandate from the Ministry of Health for the researchers and to a lack of synchronization between knowledge production and policy-making. | stakeholders, both researchers and policy makers planning to update clinical guidelines in LIC may need methodological support to overcome the expected barriers. |
| Guleid et al., 2022 | assessed the usefulness of these activities to identify the facilitators and barriers to KT and suggest actions that facilitate KT in similar setting | Qualitative study | the KT activities increased evidence availability and accessibility, enhanced policy-makers’ motivation to use evidence, improved capacity to use research evidence and strengthened relationships. Policy-makers shared that a key facilitator of this was the knowledge products shared and the regular interaction with researchers. A key barrier was the timeliness of generating evidence, which was exacerbated by the pandemic. | They felt it was important to institutionalise KT to improve readiness to respond to public health emergencies. This study provides a real-world example of the use of KT during a public health crisis. |
| Gyamfi et al., 2020 | assessed nurses’ perceptions of a recently completed Task Shifting Strategy for Hypertension control (TASSH) trial in Ghana, and facilitators and challenges to TASSH implementation. | Qualitative | Assessing stakeholders’ perception of the TASSH implementation process guided by CFIR is crucial as it provides a platform for the nurses to thoroughly evaluate the task shifting program, while considering the local context in which the program is implemented. | The feedback from the nurses informed barriers and facilitators to implementation of TASSH within the current healthcare system, and suggested system level changes needed prior to scale-up of TASSH to other regions in Ghana with potential for long-term sustainment of the task shifting intervention |
| Hyder et al., 2007 | to provide the background, conceptual framework, and key research directions for empirical activities focused on the research-policy interface in LMICs settings | Review of policy interface process | The interface can be strengthened through such analysis leading to potential improvements in population health in LMICs settings. | Health system development cognizant of the myriad factors at the research-policy interface can form the basis for innovative future health systems. |
| Hutchinson et al., 2011 | to understanding and explaining how and why certain evidence makes its way into policy and practice and what local factors influence this process. | Review | Key lessons about policy development are drawn from the research evidence on co-trimoxazole prophylaxis, as such lessons may prove helpful to those who seek to influence the development of national policy on isoniazid preventive therapy and other treatments. Researchers are encouraged to disseminate their findings in a manner that is clear, but they must also pay attention to how structural, institutional and political factors shape policy development and implementation | Mainstreaming policy analysis approaches that explain how local factors shape the uptake of research evidence can provide an additional tool for researchers who feel frustrated because their research findings have not made their way into policy and practice. |
| Henriksson et al., 2019 | To investigate how the use of this evidence affects decision-making in the planning process and how stakeholders in the planning process | Mixed method study | evidence and a structured process for its use to prioritise activities and make decisions in the planning process at the district level helped systematize the planning process. | the reported limited decision and fiscal space, inadequate funding and high dependency on donor funding did not always allow for the use of district-specific evidence in the planning process |
| Hedt-Gauthier et al., 2017 | to implement health systems strengthening initiatives inclusive of research capacity building. | Mixed method research | many common challenges for RCB, such as adequate resources and local and international institutional support, were not identified as major challenges for these projects. | provide adequate and flexible funding for RCB activities and for institutions to offer a spectrum of RCB activities to enable continued growth, provide adequate mentorship for trainees and systematically monitor RCB activities. |
| Hawkes et al., 2016 | establishing a network to disseminate evidence directly to policy makers, | Review | strengthening the capacity of individuals and organizations is an important but likely insufficient step in ensuring the use of evidence/data in policy-cycles. | Sustainability of evidence-informed policy making requires strengthening institutional capacity, as well as understanding and addressing the political environment, and particularly the incentives facing policy makers |
| Hyder et al., 2011 | to understand the perspectives and attitudes of policy-makers towards the use and impact of research in the health sector in low- and middle-income countries. | Mixed method (qualitative study and review) | barriers to evidence-based policy-making included poor communication and dissemination, lack of technical capacity in policy processes, as well as the influence of the political context. Policy-makers had a variable understanding of economic analysis, equity and burden of disease measures, and were vague in terms of their use in national decisions. | Policy-maker recommendations regarding strategies for facilitating the uptake of research into policy included improving the technical capacity of policy-makers, better packaging of research results, use of social networks, and establishment of fora and clearinghouse functions to help assist in evidence-based policy-making |
| Inguane et al., 2020 | to address these gaps and to identify challenges and facilitators to evidence-based decision-making | Qualitative study | limited demand for evidence, limited capacity to use evidence, and lack of trust in the available evidence. By contrast, access to evidence, and availability of evidence were viewed positively and seen as potential facilitators. Organizational capacity for the demand and use of evidence appears to be the greatest challenge; while individual capacity is also a barrier. Evidence-based decision-making requires that actors have access to evidence and are empowered to act on that evidence | requires alignment between those who collect data, those who analyze and interpret data, and those who make and implement decisions. Investments in individual, organizational, and systems capacity to use evidence are needed to foster practices of evidence-based decision-making for improved maternal and child health |
| Jessani et al., 2021 | explored the appropriateness of using the exploration, preparation, implementation, and sustainment (EPIS) framework for this purpose. | Evidence mapping | The mapping exercise revealed an IKT approach that was much more iterative, dynamic, and engaging than initially thought. Several constructs (phase-agnostic) remained important and stable across EPIS phases: stable and supportive funding; committed and competent leadership; skilled and dedicated IKT champions; diverse and established personal networks; a conducive and enabling policy environment; and boundary-spanning intermediaries. | Phase-agnostic constructs proved critical to ensure resilience deliberations and policies in the face of highly dynamic and changing local contexts, particularly in view of the current coronavirus disease 2019 (COVID-19) pandemic. Bridging IKT with a framework from implementation science helps to reflect on this process and can guide the development and planning of similar interventions and strategies. |
| Jha et al., 2018 | to integrate ERC best practices into governmental and non-governmental health systems for all emergencies of public health concern | Systematic review of literature | Exercises and trainings were recognized as effective strategies to identify the barriers and successes in this process of integration. Key elements to enhance information sharing and coordination across organizations included the creation of networks, task-forces and committees across disciplines, organizations and geographic areas. Engagement of local stakeholders | related to the WHO research questions, demonstrating the need for research in these areas. To facilitate an accurate identification of the gaps, the authors suggest integrating current findings with case studies across the WHO regions to better understand the specific evidence that is needed in practice across the multitude of ERC functions. |
| Kalibala & Nutley, 2019 | , recognized this gap and developed an approach to include the end data users in the research process from inception to final results dissemination | Review | there is limited evidence that, when researchers actively promote utilization of research findings stakeholders use such findings for decision making in LMIC. A common barrier for research uptake in LMIC is that researchers focus on passive dissemination of final findings as the primary vehicle to affect research uptake. | we make recommendations for active facilitation of research uptake using emerging lessons from SOAR’s RU process that focuses on ongoing engagement of stakeholders throughout the life of the study |
| Kapiriri, 2020 | To examine how Ugandan health policy-makers define and attribute value to the different types of evidence; | Narrative review | highlighted differences in the perceptions, access to, and use of evidence in priority setting in the different programs. The strong infrastructure in place to support for the access to and use of evidence in the politicized and donor supported programs should be leveraged to support the availability and use of evidence in the relatively under-resourced programs. | Further research could explore the impact of unequal availability of evidence on priority setting between health programs within the HCS. |
| Khalid et al., 2020 | To assess approach to develop a conceptual framework that outlines the strategies that leverage the facilitators and address the barriers to evidence use in crisis zones | Systematic review | a conceptual framework that focuses on evidence use in crisis zones examined through the lens of four systems – political, health, international humanitarian aid and health research. Within each of the four systems, the framework identifies the most actionable strategies that leverage the facilitators and address the barriers to evidence use. | new conceptual framework that outlines strategies that leverage the facilitators and address the barriers to evidence use in crisis zones within different systems. This study expands on the literature pertaining to evidence-informed decision-making. |
| Koon et al., 2013 | conducted to identify examples of embedded HPSR used to inform decision-making in LMICs. | Review | Our findings suggest that four qualities influence embeddedness: reputation, capacity, quality of connections to decision-makers, and quantity of connections to decision-makers and others. In addition to this, the policy environment (e.g., the presence of legislation governing the use of HPSR, presence of strong civil society, etc.) strongly influences uptake. | understand which conditions are likely to enhance uptake of HPSR in LMIC health systems. This raises several important considerations for decision-makers and researchers about the arrangement and interaction of evidence-generating organizations in health systems. |
| Kredo et al., 2018 | explores national stakeholders’ perceptions of processes informing CPG development for primary healthcare in South Africa | Qualitative study | Many challenges were attributed to inadequate financial and human resources, which were perceived to hinder capacity to undertake the necessary methodological work, respond to stakeholders’ feedback, and document and share decision-making processes. Challenges were also linked to a complex web of politics, power and interests. The CPG development arena was described as saturated with personal and financial interests, groups competing for authority over specific territories and unequal power dynamics which favor those with the time, resources and authority to make contributions. | These were all perceived to affect efforts for transparency, collaboration and inclusivity in CPG development. Dedicated resources for CPG development, standardized systems for managing conflicting interests, and the development of a political environment that fosters collaboration and more equitable inclusion within and between CPG development groups are needed |
| Kumar et al., 2020 | conducted to understand the use of evidence in policy and financing decisions for large-scale community health programmes in low- and middle-income countries. | Qualitative study | We found that evidence use is limited at all levels, in part due to a perceived lack of high-quality, relevant evidence. This perception stems from two main areas: first, desire for local evidence that reflects the context, and second, much existing economic evidence does not deal with what decision-makers value when it comes to community health systems. | Beyond the evidence gap, there is limited capacity to assess and use the evidence. Elected officials also face political challenges to disinvestment as well as structural obstacles to evidence use, including the outsized influence of donor priorities |
| Langlois et al., 2019 | aims to analyses ten embedded implementation research projects in order to identify barriers and facilitators to embedding research into policy and practice | Qualitative study | main barriers to effective delivery or scale-up of health interventions identified in the research projects were inadequate financing, fragmentation of healthcare services and information systems, limited capacity of health system stakeholders, insufficient time, cultural factors, and a lack of information. Decision-makers’ experience in embedded research showed strong engagement in protocol development, moderate engagement in data collection and low engagement in data analysis. | Embedding research into policy and practice stimulates the relevance and applicability of research, while promoting decision-makers’ engagement and likelihood to use research evidence in policy-making and health systems strengthening. |
| Lavis et al., 2008 | capturing the views and experiences of many individuals who are familiar with an organization, including staff, advocates, and critics. | Qualitative study | Two organizational strengths were repeatedly cited by individuals participating in the site visits: use of an evidence-based approach and existence of a strong relationship between researchers and policymakers (which can be challenged by conflicts of interest).  Two organizational weaknesses: a lack of resources and the presence of conflicts of interest were repeatedly cited by individuals participating in the site visits. | . Strong relationships between researchers and policymakers bodes well given such interactions appear to increase the prospects for research use. The time-consuming nature of an evidence-based approach, on the other hand, suggests the need for more efficient production processes that are 'quick and clean enough. |
| Liverani et al., 2018 | explores these issues in the country setting of Cambodia, where the Ministry of Health has explicitly championed the language of evidence-based approaches to policy and planning | Mixed method study | structural arrangements that may increasingly work to facilitate the supply of health-related data and information, and their use to inform policy and planning. However, other trends and features appear to be more problematic, including gaps between research and public health priorities in the country, the fragmented nature of research activities and information systems, the lack of a national policy to support and guide the production and use of evidence for health policy, and challenges to the use of evidence for intersectoral policymaking. | continued investments to increase the supply and quality of health data and information are needed, but greater attention should be paid to the enabling institutional environment to ensure relevance of health research products and effective knowledge management |
| Malama et al., 2021 | explore the process of health research knowledge translation into policy and to identify factors that facilitate or hinder the process in Zambia. | Qualitative study | there are policy efforts to promote knowledge translation through improvement of the research macro-environment. However, the interviews showed that coordination and linkage of the knowledge creation, translation and policy-making processes remains a challenge owing to lack of research knowledge translation capacity, limited resources and lack of knowledge hubs. Emerging local research leadership and the availability of existing stock of underutilized local health research data provide an opportunity to enhance knowledge translation to feed into policy processes in Zambia. | Public health research knowledge translation into policy remains a challenge in Zambia. To enhance the uptake of research evidence in policy-making, this study suggests the need for improved coordination, financing and capacity-building in knowledge translation processes for both health researchers and policy-makers |
| Mauti et al., 2019 | aims to assess the extent to which this commitment is being translated into the process of governmental policy-making and supported by international development partners as well as non-state actors. | Qualitative study | major health challenges that are influenced by various social determinants, but the implementation of intersectoral action focusing on health promotion is still arbitrary. Many health-related collaborations exist under the concept of intersectoral collaboration, which is prominent in the country’s development framework –. Under the political stream, the study highlights that political commitment from the highest office would facilitate mainstreaming the evidence-informed policy | The budgeting process and planning for the Sustainable Development Goals were found to be potential windows of opportunity.  it is still perceived by many stakeholders as the business of the health sector, rather than a policy for the whole government and beyond. |
| Mijumbi et al., 2014 | aim to address the barriers of timeliness and relevance of evidence at the time it is needed | Rapid review | The most common requests for evidence were about governance and organization of health systems. Regular contact between the policymakers and the researchers at the response service was an important factor in response to, and uptake of the service. | Rapid response mechanisms designed to meet policymakers’ urgent needs for research evidence about health systems are feasible and acceptable to policymakers in LMICs. |
| Mogueo et al., 2022 | aims to examine from the healthcare providers’ and policymakers’ experiences and perspectives, | Qualitative study | Factors identified by healthcare providers mainly related to self-management of the disease at the organizational and individual levels, whereas policymakers reported factors chief at the central and organizational levels | Healthcare providers involved in the decision-making process for the health care delivery and have a sense of ownership and responsibility |
| Motani et al., 2019 | evaluates the extent to which EVIDENT achieved its intended activities, documents the lessons learned and draws on these lessons learned to inform future activities of EVIDENT | Cross-sectional study | Identified barriers to these processes included little experience in evidence-informed decisionmaking (EIDM), difficulties in engaging stakeholders, challenging local environments (e.g., donor influence, bureaucracy, inaccessibility to scientific research, poor internet connectivity), and limited time and funding. However, EVIDENT activities were driven by a local need for EIDM, a sheer interest and commitment to the cause, and the opportunity for the Global North and South to work together and build relationships. | strengthened partnership, capacity and visibility on EIDM in Africa. Innovative and long-term capacity-building, dedicated leadership, stakeholder engagement and sustainable financing, leadership and functional skills across the Global South, investment in stakeholder engagement, context-specific EIDM, enhanced communication and linking, and strengthening relationships with existing stakeholder organizations. |
| Mubyazi & Gonzalez-Block, 2005 | To describe the (a) role of researchers in producing evidence that influenced the Tanzanian government replace chloroquine | Qualitative study | Changing national drug policy will remain a sensitive issue that cannot be done overnight. However, to ensure that research findings are recognized and the recommendations emanating from such findings are effectively utilized, a systematic involvement of all the key stakeholders (including policy-makers, drug manufacturers, media, practitioners and the general public) at all stages of research is crucial. | It also matters how and when research information is communicated to the stakeholders. Professional organizations such as the East African Network on Malaria Treatment have potential to bring together malaria researchers, policy-makers and other stakeholders in the research-to-drug policy change interface. |
| Murunga et al., 2020 | aim of identifying gaps and informing future research and interventions | Systematic review | range of barriers at individual and institutional levels that limit their knowledge translation (KT) practice, including inadequate knowledge and skills, poor communication of research and interacting with research end-users, insufficient funding, and inadequate institutional guidelines, structures and incentives promoting KT practice. | More high-quality research on researchers’ KT capacity, practice and effective KT capacity strengthening interventions is needed. |
| Musango et al., 2012 | To explore insight on how to build on the existing convergence and to smoothen to design solutions to some of the key health financing problems | Debate articles | The relatively strong political commitment to health has not always translated into more public spending for health. Donor investment in health in LMICs still falls below commitments. There is need to explore innovative domestic revenue collection mechanisms. | inadequate funding for health is a fundamental problem, inefficient use of resources is of great concern. The current unsatisfactory state of health financing was mainly attributed to lack of clear vision; evidence-based plans and costed strategies. |
| Nabyonga-Orem et al., 2014 | explored the barriers and facilitating factors to uptake of evidence in the process of user fee abolition in Uganda and how the context and stakeholders involved shaped the uptake of evidence. | Qualitative study | The capacity of the MoH to lead the KT process was weak and the partnerships for KT were informal. The political window and alignment of the evidence with overall government discourse enhanced uptake of evidence. Stakeholders were divided, seemed to be polarized for various reasons and had varying levels of support and influence impacting the uptake of evidence. | Evidence will be taken up in policy development in instances where the MoH leads the KT process, there are partnerships for KT in place, and the overall government policy and the political situation can be expected to play a role. |
| Nabyonga-Orem et al., 2016 | to discuss the role of health policy dialogue in improving harmonization and alignment to national health policies and strategic plans, and to provide an analytical view of the critical factors in realizing a good outcome from a health policy dialogue process | Debate | Strengthening policy dialogue to support the development and implementation of robust and comprehensive national health policies and plans, as well as to improve aid effectiveness, is seen as a strategic entry point to improving health sector results. However, unbalanced power relations, the lack of contextualized and relevant evidence, the diverse interests of the actors involved, and the lack of conceptual clarity on what policy dialogue entails impact the outcomes of a policy dialogue process. The critical factors for a successful policy dialogue have been identified as adequate preparation; secured time and resources to facilitate an open, inclusive and informed discussion among the stakeholders; and stakeholders’ monitoring and assessment of the dialogue’s activities for continued learning. | Policy dialogue needs to be appreciated as a complex and iterative process that spans the whole process of policy-making, implementation, review and monitoring, and subsequent policy revisions. The existence of the critical factors for a successful policy dialogue process needs to be ensured whilst paying special attention to the peculiarities of LMICs and potential power relations, and mitigating the possible negative consequences. There is need to be cognizant of the varied capacities and interests of stakeholders and the need for capacity building, and to put in place mechanisms to manage conflict of interest. |
| Naghibi et al., 2021 | aimed to update the findings of the previous research by reviewing studies published after 2014 | Systematic review | barriers were divided into six main themes: system-level barriers, barriers related to the evidence, individual-related, communicational, resource, patient-related, and external barriers. Lack of time, support, and skills had the highest repetition, respectively. | The results of our previous study were updated, and further barriers were identified and reported. Policymakers and managers can use the results as a practical guide to expand and improve EBP and remove barriers. |
| Namisango et al., 2021 | To explore the views of policymakers regarding data availability, data gaps and preferred data formats to support policy and decision making for palliative cancer care in Nigeria, Uganda and Zimbabwe | Qualitative study | The policymakers data and information concerns are aligned to the MEASURE evaluation framework of data and information use and include; low prioritization of cancer; identifying and engaging the data user; improving data quality; improving data availability and accessibility; identifying information needs; capacity building in core competencies (e.g. skills gaps); strengthening organizational data demand and use (e.g. policy frameworks); monitoring, evaluating and communicating of data demand and use (e.g. trustworthiness of data). | Evidence of data source, challenges to their access and use, guidance on data needs for policymakers, and opportunities for better engagement between data producers, brokers and users. This framework of evidence should inform the development of strategies to improve data access and use for policy and decision making to improve palliative cancer services in participating countries with relevance to the wider region. |
| Naude et al., 2015 | to understand the policymaking process and how research evidence may contribute in South Africa and Cameroon. | Qualitative study | influenced by political structures, relationships between national and subnational levels, funding and international stakeholder agendas. Research is not a main driver of policy, but rather current contextual realities, costs, logistics and people (clinicians, NGOs, funders) influence the policy, and research plays a part. Research evidence is frequently perceived as unavailable, inaccessible, ill-timed or not applicable. EIDM is regarded as necessary in South Africa but is less well understood in Cameroon. Insufficient time and capacity were hindrances to EIDM in both countries. Good relationships between researchers and policymakers may facilitate EIDM. Policymaking is complicated, lengthy and mostly done at national level. | Researchers should have a good understanding of the policymaking environment if they want to influence it. Greater interaction between policymakers and researchers is perceived as beneficial when formulating research and policy questions as it raises researchers’ awareness of implementation challenges and enables the design of tailored and focused strategies to respond to policymakers’ needs. It can inform effective dialogue between researchers and policymakers, and contribute to enhancing use of research EIDM. |
| Norton et al., 2019 | explored its application in the context of KB decisions regarding evidence for use in LMICs. | Cross-sectional study | Application of the TDF involved challenges related to overlapping constructs, retrospective use, and complexities of global health settings and relevant knowledge. Codes needed to be added or adapted to account for how KBs’ internal reflections on external factors influenced their actions in selecting evidence to share and use, and the decisions they made during the process. Four themes of the rationale for changing the TDF were identified during analysis, namely Influences from beyond the organization, Knowledge Selection as a Process, Access and Packaging of Knowledge, and Fit for Use. | Theories of individual behavior, such as those in the TDF, can enhance understanding of the decisions made by actors such as KBs along dissemination and knowledge translation pathways. Understanding how KBs reflect on evidence and interact with their environment has the potential for improving global dissemination efforts and South-south exchange of implementation evidence. |
| Okedo‑Alex et al., 2021 | assessed the status of HPSR domestic funding and advocacy strategies for improving HPSR funding in Nigeria. | Mixed method study | Lack of funding, bureaucratic bottlenecks, political and policy transitions, and corruption. Potential opportunities centered on existing policy documents and emerging private sector willingness to fund health research. Multi-stakeholder advocacy coalitions, continuous advocacy and researcher skill-building on advocacy with active private sector involvement were the strategies proffered by the participants. Pre-workshop, understanding of the meaning of HPSR had the highest mean ratings while knowledge of budgeting processes and use of legal action to enable opportunities for budget advocacy for HPSR funding had the lowest mean ratings. Following the capacity-building workshop, all knowledge and understanding parameters markedly improved from 12.5%–71.0%. | recommend the deployment of these identified strategies and wider national and regional stakeholder engagement towards prioritizing and improving domestic funding for HPSR. |
| Orem et al., 2012 | objective of this research is to elaborate a Middle Range Theory (MRT) of KT in Uganda that can also serve as a reference for other LMICs | Qualitative  Study | most common emerging facilitating factors could be grouped under institutional strengthening for KT, research characteristics, dissemination, partnerships and political context. The analysis of interviews, however, showed that policymakers and researchers ranked institutional strengthening for KT, research characteristics and partnerships as the most important. | coordinate and disseminate research, the separation of roles between researchers and policymakers, and the role of the community and civil society in KT. It provides a framework that can be used in empirical research of the process of KT on specific policy issues. |
| Paci et al., 2021 | To review and meta-analysis the evidence about the prevalence of barriers to evidence-based practice (EBP) reported in physiotherapy | Systematic reviews | lack of time, lack of access, lack of statistical skills, Lack of skills, lack of generalizability, lack of support and lack of interest are less frequent were declared as barriers. | Organizational issues and methodological skills seem key issues to allow the implementation of EBP, suggesting the need to adopt or enhance organizational and training strategies to facilitate the implementation of the EBP. |
| Poursheikhali et al., 2022 | aims to assess the HRS in terms of stewardship functions and highlight the enhancement points. | Systematic reviews | Analysis of the findings identified eight main themes. The most critical challenges were the lack of an integrated leadership model and a shared vision among different HRS stakeholders. Their scope and activities were often contradictory, and their role was not clarified in a predetermined big picture. The other challenges were legislation, priority-setting, monitoring and evaluation, networking, and using evidence as a decision support base. Stewardship functions are not appropriately performed and are considered the root causes of many other HRS challenges in Iran | Formulating a clear shared vision and a work scope for HRS actors is critical, along with integrating all efforts towards a unified strategy. Policy-makers and senior managers need to embrace and use evidence, and effective networking and communication mechanisms among stakeholders redesigning the processes, regulations and rules to promote transparency and accountability within a well-organized and systematic framework need to be enhanced. |
| Price et al., 2016 | aim of this article is to recommend strategies for bridging this divide between production and utilization of NHA data in low-resource settings. | Review | The focus then turns to the development and application of strategies to assist LMICs in ‘unlocking’ the hidden value of their NHA. The article draws on the example of Fiji, a country currently attempting to integrate their NHA data into policy formulation, despite minimal resources, training and familiarity with economic analysis of health systems. | Simple, low-cost recommendations such as embedding health finance indicators in planning documents, a user-friendly NHA guide for evaluating local health priorities, and sharing NHA data for collaborative research have helped translate NHA from raw data to evidence for policymaking. |
| Puchalski Ritchie et al., 2019 | to identify barriers and facilitators to utilization of EBCAs, to identify priority targets for development and to understand the process of care in the TASH-ED to inform implementation planning | Qualitative study | Lack of medication, equipment and human resources were identified as the primary barriers to use of EBCAs in the TASH-ED. Support from leadership and engagement of stakeholders outside the ED where EBCAs were believed to be less well accepted were identified as essential facilitators to implementation of EBCAs in the TASH-ED. | perceived need for EBCAs tailored to the TASH-ED setting to support uptake of evidence-based care into routine practice for common clinical presentations. Barriers and facilitators provide information essential to development of both context-appropriate EBCAs and plans for their implementation in the TASH-ED. |
| Kalbarczyk et al., 2021 | conducted a review of the grey and published literature to identify country health priorities and established barriers and facilitators for KT | Systematic review complemented by KII | support many well-documented barriers including lack of time, skills and institutional support to conduct KT. Three additional institutional drivers emerged around soft skills and the complexity of the policy process, alignment of incentives and institutional missions, and the role of networks. Participants reflected on often-lacking soft-skills needed by researchers to engage policy makers. Continuous engagement was viewed as a challenge given competing demands for time and lack of institutional incentives to conduct KT. | Strong networks, both within the institution and between institutions, were described as important for conducting KT but difficult to establish and maintain. Attention to the cross-cutting themes representing barriers and facilitators for both individuals and institutions can inform the development of capacity building strategies that meet readiness needs. |
| Rosenbaum et al., 2011 | To describe how the SUPPORT collaboration developed a short summary format for presenting the results of systematic reviews to policy-makers in LMICs. | Review | Policy-makers liked a graded entry format (i.e., short summary with key messages up front). They particularly valued the section on the relevance of the summaries for LMICs, which compensated for the lack of locally-relevant detail in the original review. Some struggled to understand the text and numbers. Three issues made redesigning the summaries particularly challenging: participants had a poor understanding of what a systematic review was; expected information not found in the systematic reviews | Presenting evidence from systematic reviews to policy-makers in LMICs in the form of short summaries can render the information easier to assimilate and more useful, but summaries must be clear and easy to read or scan quickly. They should also explain the nature of the information provided by systematic reviews and its relevance for policy decisions |
| Li R et al., 2017 | to identify those stakeholders, whose capacity needs will vary along the evidence-to-policy continuum. | Review | approach to capacity building; rather a spectrum of activities that recognizes the roles and skills of all stakeholders, formal and informal training, networking and engagement, and support through collaboration on projects, should be flexibly employed (and tailored to specific needs of each country) to support institutionalization of evidence-informed priority-setting | capacity building should be a two-way process; those who build capacity should also attend to their own capacity development in order to sustain and improve impact. |
| Schleiff et al., 2020 | limited evidence about what works to strengthening capacity at individual and institutional levels within countries has been reported, and inconsistent use of evidence to inform policy-making is a persistent challenge and concern. | Comparative analysis | The cross-cutting themes included leadership and political will, incentives and resources, infrastructure and access to health data, designated structures and processes, interaction and relationships, and capacity strengthening and engagement. While each case country’s context and experience were different, common enablers and barriers surfaced across each of these themes, but also recognizing the need for other stakeholders, including researchers, donors and civil society, to serve as essential collaborators in order to strengthen evidence uptake. | Substantial and sustained investment in research capacities, able leaders and stronger engagement of civil servants are needed to further this progress and strengthen processes of health decision-making. Increase evidence uptake and strengthening supportive systems. Establishing and strengthening necessary structures and the relationships that underpin them takes time as well as resources. Guide and support advocacy to increase domestic funding for health research, researchers have the capacity and support to collaborate |
| Shidhaye et al., 2015 | main elements and features of a mental health care delivery platform and its delivery channels. | Review | Key strategies for effective translation of evidence into action include collaborative stepped care, strengthening human resources, and integrating mental health into general health care. | Principles and strategies using a platform-wide approach, policy makers need to engage with a wide range of stakeholders and make use of the best available evidence in a transparent manner. |
| Shroff et al., 2017 | brings together elements from both sides to analyses institutional capacity for the generation of HPSR and the use of evidence more broadly in LMICs. | Review | Findings from the survey of research institutions identified the absence of core funding, the lack of definitional clarity and academic incentive structures for HPSR as significant constraints. The survey of Ministries of Health identified a lack of locally relevant evidence, poor presentation of research findings and low institutional prioritization of evidence use as significant constraints to evidence uptake. In contrast, improved communication between researchers and decision-makers and increased availability of relevant evidence were identified as facilitators of evidence uptake. | The findings make a case for institutional arrangements in research that provide support for career development, collaboration and cross-learning for researchers, as well as the setting up of institutional arrangements and processes to incentivize the use of evidence. Build institutional capacity through engaging multiple stakeholders in identifying and maintaining incentive structures, improving research training, and developing stronger tools for synthesizing non-traditional |
| Singh et al., 2023 | to understand how knowledge about collaborative working can be translated into policy and practice in the context of LMICs. | Perspective | concept of co-design, co-production, and co-creation in health and the potentiality and challenges of using co-design in health services research in LMICs. Despite the challenges, the co-design research has considerable potential to encourage the meaningful engagement of service users and other stakeholders in developing, implementing, and evaluating real-world solutions in LMICs | It is essential to balance power dynamics in a co-design process through mutual recognition and respect, participation. The inclusive and collaborative approach to working is complex due to existing rigid hierarchical structures, socio-cultural beliefs, political interference and working practices. |
| Tangcharoensathien et al., 2022 | assesses HPSR capacity to generate evidence and inform policy in Ethiopia and Ghana. | Mixed method study | Both countries have limited capacity to generate HPSR evidence, especially in terms of mobilizing adequate funding and retaining a critical number of competent researchers who understand complex policy processes, have the skills to influence policy, and know policy makers’ demands for evidence. Common challenges are limited government research funding, rigidity in executing the research budget, and reliance on donor funding or budget for health research that might not respond to national health priorities | Strengthen researchers’ capacity and enhance their opportunities to know policy actors; engage with the policy community; and identify and work with policy entrepreneurs, who have attributes, skills, and strategies to achieve a successful policy. Mobilize local and international funding to strengthen HPSR capacities as well as address challenges with recruiting and retaining a critical number of talented researchers. |
| Unadkat et al., 2021 | explored the self-reported knowledge, attitudes, practices and barriers of evidence-based practice among resident physicians in a tertiary teaching hospital. | Mixed method study | The most common barriers faced by residents were lack of time, lack of EBM skills and patients’ unawareness about evidence-based making. From the qualitative study, residents demonstrated good knowledge and support of EBM but practice remained relatively poor. Barriers to EBM were characterized by lack of motivation, time, skills and resources, patient overload and fear of challenging consultants. | There was good understanding and support of EBM among residents, though challenges were experienced in regards to practice of EBM because of lack of time and skills. Resources should be allocated towards integrating EBM into cultivate critical thinking skills at an early stage before transition into residency. |
| Uzochukwu et al., 2016a | reports the experiences of a research group in a Nigerian university when seeking to ‘do’ GRIPP, and the important features and challenges of this process within the African context. | Qualitative study | stakeholders’ request for evidence to support the use of certain strategies or to scale up health interventions; policymakers and stakeholders seeking evidence from researchers; involving stakeholders in designing research objectives and throughout the research process; and facilitating policy maker-researcher engagement in finding best ways of using research findings to influence policy and practice and to actively disseminate research findings to relevant stakeholders and policymakers. | The challenges to research utilization in health policy found were to address the capacity of policy makers to demand and to uptake research, the communication gap between researchers, donors and policymakers, the management of the political process, the lack of willingness of some policy makers to use research, the limited research funding and the resistance to change. |
| Uzochukwu et al, 2016b | To explore health policy and systems research assets and needs of different stakeholders, and their perspectives | Cross-sectional study | lack of awareness and funding were identified as barriers to strengthening HPSR+A in Nigeria. Policymakers were not aware of the availability of research findings that could inform the policies they make nor where they could find them. | Policymakers were not aware of the availability of research findings that could inform the policies. They also appeared unwilling to go through the rigors of reading extensive research reports. |
| VanDevanter et al., 2020 | present findings from a post-implementation qualitative assessment of factors influencing effective implementation and program sustainability. | Qualitative study | The primary challenges to sustainability were competing priorities that are driven by the Ministry of Health and may result in fewer resources for TDT compared with other health programs. However, providers and VHWs suggested several options for adapting the intervention and implementation strategies to address challenges and increasing engagement of local government committees and other sectors to sustain gains. | Sustaining gains in practice improvement and clinical outcomes will require strategies that include ongoing engagement with policymakers and other stakeholders at the national and local level, and planning for adaptations and subsequent resource allocations. |
| Vania and Randall, 2016 | to determine whether the implementation of an organ donor registry is a feasible and appropriate policy option to enhance cadaver organ donation rates in a lower-income country | Qualitative study | Findings indicate that organ donation in India is a complex issue due to low public awareness, misperceptions of religious doctrines, the need for family consent, and a nation-wide focus on disease control. Key informants cite social, political, and infrastructural barriers to the implementation of an organ donor registry, including widely held myths about organ donation, competing health priorities, and limited hospital infrastructure. | balance international pressures to improve overall population health with the desire to also enhance individual health. Implementing an organ donor registry in Mumbai is not a feasible or appropriate policy option in India’s current political and social environment, as the barriers, identified through the 3-i framework lens |
| Vanyoro et al., 2019 | aim to influence health systems planning, costing, policy and implementation. | Review | there is a recognition in policy studies more broadly about the role of co-production, collective ownership and the value of localized HPSR in the evidence-to-policy discussion, ‘ownership’ of research at country level is a research uptake catalyst that needs to be further emphasized, particularly in the HPSR context. | Participatory or community-initiated research and emergent/responsive research processes, ownership policymakers, healthcare practitioners/ managers or community members who are directly affected by health problems connects research and decision-making and, creating pathways to impact |
| Varallay et al., 2020 | To design to guide the systematic evaluation of embedded IR | Mixed method implementation research | collaborative research approach influences programme improvement, it outlines expected processes and intermediate outcomes. It also highlights constructs with which to assess ‘embeddedness’ as well as critical contextual factors. The framework is intended to provide a structure by which to systematically examine such embedded research initiatives, proposing three key stages of evidence-informed decision-making – co-production of evidence, engagement with research, and enactment of programme changes. | Rigorous evaluation of embedded IR is needed to build the evidence on its processes and effectiveness in influencing decision-making. The evaluation framework presented here addresses this gap with consideration of the complexity of such efforts. Its applicability to similar initiatives is bolstered by virtue of being founded on real-world experience; its potential to contribute to a nuanced understanding of embedded IR is significant. |
| Verboom & Baumann, 2022 | aimed to comprehensively map the global, peer-reviewed qualitative literature on the use of research evidence in health policy-making | Systematic review | evidence that theory-driven and explanatory (eg, comparative case study) designs are becoming more common in this literature. Investigations of the barriers and facilitators related to evidence use constitute a large proportion but by no means a majority of the work in this area. | Provides a bird’s eye mapping of the peer reviewed qualitative research on evidence-to-policy processes, and has identified key features of – and gaps within – this body of literature that will hopefully inform, and improve, research in this area moving forward |
| Votruba et al., 2020 | identified a gap in frameworks on agenda setting and actionability, and pragmatic, effective tools to guide action to link research and policy are needed | Mixed method study | EVITA 1.1 consists of six core components [advocacy coalitions, (en)actors, evidence generators, external influences, intermediaries and political context] and four mechanisms (capacity, catalysts, communication/relationship/partnership building and framing). EVITA 1.1 is novel and unique because it very specifically addresses the mental health research–policy process in LMICs and includes policy agenda setting as a novel, effective mechanism. | Based on a thorough methodology, and through its specific design and mechanisms, EVITA has the potential to improve the challenging process of research evidence translation into policy and practice in LMICs and to increase the engagement and capacity of mental health researchers, policy agencies/planners, think tanks, NGOs and others within the mental health research–policy interface. |
| Votruba et al., 2021 |  |  | research translation to the policy agenda occurs in a com- plex, fluid system which includes multiple “research clouds”, “policy spheres” and other networks; mental health research policy agenda-setting is based on key individuals and intermediaries and their interrelationships; and key challenges and strategies for successful research to policy agenda impact are known, but are frequently not strategically implemented, such as including all stakeholders to overcome the policy implementation gap. Our data also suggest that behavioural science methods can be strategically applied to support knowledge translation to policy agenda-setting. | the EVITA framework is useful for understanding and improving mental health research policy interrelationships to support evidence uptake to the policy agenda, and that behavioural science methods are effective support mechanisms. The revised EVITA 2.0 framework therefore includes behavioural insights, for improved mental health policy agenda-setting in LMICs. More research is needed to understand whether EVITA can be applied to other LMICs and to high-income contexts. |
| Witter et al., 2019 | examines policy transfer and evidence use linked to it in LMICs and inform international investments in improved learning across health systems. | Comparative study | agency of local actors and the importance of developing national and sub-national institutions for gathering, filtering and sharing evidence. Developing demand for and capacity to use evidence appears more important than augmenting supply of evidence, although specific gaps in supply were identified. | The findings also highlight the importance of the local political economy in setting parameters within which evidence is considered and the need for a conceptual framework for health system learning |
| Witter et al., 2017 | to assess whether the 3DE model was successful in supporting and increasing evidence-based policymaking, building capacity and changing behaviour of Ministry staff. | Mixed method study | The importance of supporting evaluative thinking and capacity within wider institutions, of understanding the political economy of evidence use and its uptake, and of allowing for some flexibility in terms of programme targets. Fixating on one type of evidence is unhelpful in the context of institutions like ministries of health, which require a wide range of evidence to plan and deliver programmes. | having success tied to indicators, such as number of ‘policy decisions made’, provides potentially perverse incentives and neglects arguably more important aspects such as incremental programmatic adjustments and improved implementation. |
| Yamey, 2012 | aimed to: (a) explore the barriers that have impeded such scale-up in LMICs, and (b) lay out an “implementation research agenda”—a series of key research questions that need to be addressed in order to help overcome such barriers. | Qualitative study | Factors impeding the success of scale-up that emerged from the key informant interviews, and which are areas for future investigation, include: complexity of the intervention and lack of technical consensus; limited human resource, leadership, management, and health systems capacity; poor application of proven diffusion techniques; lack of engagement of local implementers and of the adopting community; and inadequate integration of research into scale-up efforts. | Key steps in expanding the evidence base on implementation in LMICs include studying how to: simplify interventions; train “scale-up leaders” and health workers dedicated to scale-up; reach and engage communities; match the best delivery strategy to the specific health problem and context; and raise the low profile of implementation science. |
| Yamey et al., 2016 | To scaling up the use of existing evidence-based child health interventions, including those to prevent and treat tuberculosis | Systematic Review | the coverage rates of these interventions in low- and middle-income countries remain very low; and the gap between knowledge and action on PTB—the “know-do gap”—has been a major obstacle to progress in scaling up the use of existing evidence-based child health interventions, including those to prevent and treat PTB. | The evidence-based approaches to narrowing this gap have become known as knowledge transfer and exchange (KTE). We hope that our proposed research agenda stimulates further debate and discussion on research priorities to soon bend the curve of PTB mortality. |
| Orgill et al., 2012 | To outline a translational framework for public health research | Review | institutionalizing effective interventions to that of improving population health by influencing both individual and collective determinants of health. recognizing that many types of research may contribute to the shaping of policy, practice and future research. | propose a research agenda to advance the field and argue that resources for 'applied' or 'translational' public health research should be deployed across the framework, not reserved for 'dissemination' or 'implementation' |
| Cairney et al., 2017 | To exploring the frustrating experiences of scientists and identifying the need for better evidence to reduce policymaker uncertainty | Systematic review | identify two important dilemmas, for scientists and researchers, that arise from our initial advice. Involving effective actors combine evidence with manipulative emotional appeals, adapting multi-level policymaking, prioritize evidence-based policymaking, co-production of policy between local public bodies, interest groups, and service users are crucial to influence the policy agenda | successful engagement in 'evidence-based policymaking' requires pragmatism, combining scientific evidence with governance principles, and persuasion to translate complex evidence into simple stories and to maximize the use of scientific evidence in health and public health policy, researchers should recognize the tendency of policymakers. |
| Yearwood, 2018 | To assess the model’s logic and evaluated whether the expected outcome was achieved | Mixed method study | The inclusion of evidence briefs, stakeholder dialogues, a research database, and training programs for policymakers in the model was validated. Respondents also reported their intention to act on research evidence to which they were exposed. | initial validation of evidence as a consolidated strategy to strengthen the application of research in policy in the Caribbean. |
| Armstrong, et al., 2013 | To describes the design of and implementation plan for a knowledge translation intervention for public health decision making in local government | Mixed method study | effective and promising strategies to increase access to research evidence require an integrated intervention of skill development, access to a knowledge broker, resources and tools for evidence-informed decision making, and networking for information sharing, comprising workforce development, access to evidence, and regular contact with a knowledge broker to increase access to intervention evidence; develop skills in appraisal and integration of evidence; strengthen networks | It provides significant insights into how practitioners might engage with evidence in public health decision making. While this intervention model was designed for the local government context, it is likely to be applicable and generalizable across sectors and settings. |
| Compbell et al., 2009 | To explored the views and practice of policy makers and researchers regarding the use of evidence in policy, | Cross-sectional mixed method study | Policy makers reported rarely using research to inform policy agendas or to evaluate the impact of policy; research was used more commonly to inform policy content. Most researchers reported that their research had informed local policy, mainly by increasing awareness of an issue. Policy makers reported difficulty in accessing useful research syntheses, and only a third of researchers reported developing targeted strategies to inform policy makers of their findings. | increasing the use of research evidence in policy; however, both groups reported a high level of involvement by policy makers in research. four strategies to assist in increasing the use of research in policymaking research findings more accessible to policymakers; increasing opportunities for interaction between policymakers and researchers; addressing structural barriers such as research receptivity in policy agencies and a lack of incentives for academics to link with policy; and increasing the relevance of research to policy |
| Fekadu et al., 2021 | to describe the core processes of excellence as a prerequisite to establishing academic centre of excellence in Africa | Hybrid high-level review | Participatory leadership encourages innovations, *future-oriented* centres of excellence and rely on expertise from different disciplines and ‘engaged’ scholarship. This multidisciplinary leads to improved research productivity and enhances the production of problem-solving innovations. | Participatory leadership, knowledge management, and inter-disciplinary collaborations are prerequisites to establishing academic centre of excellence in Africa. |
| Petersen et al., 2017 | to identify key governance challenges, needs and potential strategies that could facilitate adequate integration of mental health into primary health care settings in LMICs | Multi-country study | strengthening capacity of managers, mainly key aspects of the essential health system building blocks to promote responsiveness, efficiency and effectiveness; developing workable mechanisms for inter-sectoral collaboration, as well as community and service user engagement; and developing innovative approaches to improving mental health literacy and stigma reduction. | Inadequate financing emerged as the biggest challenge for good governance. the need for overall good governance of a health care system, this study identifies a number of specific strategies to improve governance for integrated mental health care in low- and middle-income |
| Sumner et al., 2011 | To conceptual issues of what determines research impact and policy change | Review | identify aspects of the policy landscape and drivers of policy change commonly occurring across multiple sectors and studies to create a framework that influencing uptake and the ways in which researchers can actively influence the policy landscape and promote research uptake through their policy engagement actions and strategies. | conclude by highlighting the need for continued multi-sectoral work on understanding and measuring research uptake and for prospective approaches to receive greater attention from policy analysts. |
| Zhou et al., 2018 | to identify the transition and implementation challenges of mental health policies | Systematic review | policy domains were summarized: service organizing, service provision, service quality, human resources, legislation and human rights, advocacy, administration, surveillance and research, and financing and budgeting. were mainly related to service organizing and service provision; for MLICs, more severe implementation problems converged on financing and budgeting, administration and human resources. | funding, human resources and administration. Therefore, future efforts should not only be made on helping MLICs developing mental health policies, but also on promoting policy implementation under MLICs’ local context |
| Jenkins et al., 2011 | describes the facilitation over many years by a WHO Collaborating Centre, of sustainable mental health developments in Zanzibar, | Impact evaluation study | The policy and legislation have resulted in enhanced mental health activities over the ensuing decade, within a setting of extreme low resource. However, advances ebb and flow and continued efforts are required to maintain progress and continue mental health developments. Lessons learnt have informed the development of mental health policies in neighbouring countries. | A multi-faceted and comprehensive programme can be effective in achieving considerable strengthening of mental health programmes and services even in extremely low resource settings, but requires sustained input and advocacy if gains are to be maintained and enhanced. |
| Bowsher et al., 2019 | to strengthen health research capacity in conflict-affected countries and regions | Narrative review | lobal imbalances in research capacity, with implications for the type and priorities of research produced, equity within epistemic communities and the development of sustainable research capacity in LMICs. There is more evidence on health research capacity strengthening in general, from which several key enablers emerge: adequate and sustained financing; effective stewardship and equitable research partnerships; mentorship of researchers of all levels; and effective linkages of research to policy and practice. | Strengthening health research capacity in conflict-affected areas needs to occur at multiple levels to ensure sustainability and equity. Capacity strengthening interventions need to take into consideration the dynamics of conflict, power dynamics within research collaborations, the potential impact of technology, and the wider political environment in which they take place. |
| Glasgow et al., 2007 | To summarizes key factors that have interfered with translation of research to practice and what public health researchers | Review | The need to address context and to utilize research, review, and reporting practices that address external validity issues-such as designs that focus on replication, and practical clinical and behavioral trials-are emphasized. | Greater attention is needed to connectedness across program levels and components. that can help accelerate translation of research to practice and policy. |
| Reed et al., 2018 | to advance empirical and theoretical understanding of the reality of making and sustaining improvements in complex healthcare systems | Qualitative study | ‘act scientifically and pragmatically’ – knowledge of existing evidence needs to be combined with knowledge of the unique initial conditions of a system, and interventions need to adapt as the complex system responds and learning emerges about unpredictable effects; ‘embrace complexity’ – evidence-based interventions only work if related practices and processes of care within the complex system are functional, and evidence-translation efforts need to identify and address any problems with usual care, recognising that this typically includes a range of interdependent parts of the system; and ‘engage and empower’ – | evidence translation and system navigation require commitment and insights from staff and patients with experience of the local system, and changes need to align with their motivations and concerns. recognising how agency, interconnectedness and unpredictability influences evidence translation in complex systems, the ‘simple rules’ have potential to provide a common platform for academics, practitioners, patients and policymakers to collaborate. |
| Gilson et al., 2011 | To assess the building the Field of Health Policy and Systems Research: Social Science Matters | Review | The criticisms that Health Policy and Systems Research (HPSR) is too context specific, does not offer clear lessons for policy makers, and is not rigorous are partly a reflection of differences in knowledge paradigms between those with predominantly clinical, biomedical, and epidemiological backgrounds, underpinned by a positivist paradigm, and those with social science backgrounds underpinned by a relativist paradigm. | Health policies and systems are complex social and political phenomena, constructed by human action rather than naturally occurring. Relativist social science perspectives are, therefore, recognize that all phenomena are in essence constructed through human behavior and interpretation, and rich understanding of context; supporting policy learning; and enhancing research rigor and quality |
| Chan et al., 2015 | dissemination and implementation research in behavioral medicine | Review | resource-poor and developing regions of the world where the difference in health systems, languages, and cultures very significantly influences the translation of evidence into policy and practice. Moreover, demonstrating the broader societal and economic value of behavioral interventions in settings where they are implemented can further support the sustainability, uptake, and implementation of these findings in other settings and contexts. | address dissemination, implementation, and translation issues in both developed and developing countries. Specifically, the learnings from the application of many and varied theories and research methodologies are very relevant for bridging the current division between research findings and their translation and uptake into policy and practice. |
| Brownson et al., 2009 | To describe the key domains of evidence-based policy and tracking outcomes | Review | process, to understand approaches to enhance the likelihood of policy adoption; content, to identify specific policy elements that are likely to be effective; and outcomes, to document the potential impact of policy. | Actions to further evidence-based policy include preparing and communicating data more effectively, using existing analytic tools more effectively, conducting policy |
| Gaym et al., 2008 | The systems approach begins with a situation analysis of the existing national health research system. | Narrative review | The researcher profile, disease and health focus, publication audit, dissemination modalities and international collaboration of research institutes are outlined. The number of health researchers, research institutes and volume of research output so far is small. Detailed laws and regulations pertaining to health research are not enacted and governance of health research is not clearly articulated. There are global, regional and programmatic inequities in the national health research arena. | research funding and financing agencies; strengthening public-private partnerships in health research; maintaining national and international commitments towards research financing; increased incentives provided to researchers thereby curbing brain drain; attracting young scientists to a career of research and increasing capital expenditure in order to establish research institutes. |
| Bazalgette, 2020 | To establish an evidence translation center | Narrative review | Evidence centres are intermediaries that facilitate exchange of idea to influence individual users, professional bodies and organizations to use evidence in their decision-making. | This means that a relatively complex chain of events will need to occur before positive societal impacts can be observed. |
| FHI, 2012 | To introduce evidence to policy strategies | Program document | set of eight strategies to help close the gap between research and practice. The strategies are based on a growing body of evidence, theoretical frameworks, case studies and published guidance. | The strategies explain how to plan, implement and disseminate research to facilitate its translation into practice, and most effective ways to incorporate research results into policies and programs. |
| Elliott, 2019 | To explain the contributions to improving health care and, more specifically, healthcare as practiced through laboratory medicine | Narrative review | health practitioner may consider in making decisions about testing and treatment options. Understanding their sometimes-competing priorities, may help you to discuss your testing options with your health practitioner and make informed choices to improve your care | making informed decisions about your or a loved one’s health care as well as additional resources. |
| Abu-Odah et al., 2022 | To explore policymakers’ perspectives on health care system, an essential step to developing a palliative care program | Qualitative study | The current health care services provided to Palestinian patients with life-limiting illnesses and their families are not comprehensive, and are limited to symptom management, however, the development goals are not clearly defined, and the plan’s capabilities are inadequate. Several challenges to the provision of health care were found to relate to issues of education and training, the allocation of funding, and the availability of medications. | Integrating, establishing capacity building program to overcome the shortage of experts. Developing policies aligned with national laws could help enhance health services to patients and their families and resolve several challenges. Cooperating with national and international institutions in seeking funding could boost evidence to policy |
| Buse et al., 2008 | The aim of the paper is to generate debate to assist in resolving the myriad challenges inherent in prospective policy analysis. | Hybrid review | provides an outline approach of how researchers might work together with advocacy coalition to document and analyses the efforts of such coalitions to use policy analysis to influence the policy processes-agenda setting, policy formulation and policy implementation-in which they engage | appeals for political research which addresses the problems confronting political actors so as to guide future action-research for evidence-informed, pro-poor health policy. |
| Campos et al., 2019 | examines one aspect of implementation—the politics of policy implementation for the health sector, particularly the management of stakeholders in order to help change teams improve the chances of achieving policy objectives | Systematic review | six major categories of stakeholder groups that are likely to influence implementation: interest group politics, bureaucratic politics, budget politics, leadership politics, beneficiary politics, and external actor politics. examine the politics of these different stakeholder categories, and then present selected examples of published case studies that show the types of implementation challenges that arise for each category and how implementers can use political strategies to manage specific stakeholder groups and related political processes. | Understanding the political dimensions of implementation can help those responsible for implementation drive policy into practice more effectively. Understanding and addressing conflict, resistance and cooperation among stakeholders are key to managing the implementation process. Systematic and continuous political analysis can help decision makers and change teams improve the chances for successful implementation. |
| Oliver et al., 2006 | is to articulate a role for political analysis of public health issues, ranging from injury and disease prevention to health care reform. | Review | It begins by examining how health problems make it onto the policy agenda. Perceptions regarding the severity of the problem, responsibility for the problem, and affected populations all influence governmental responses. Next, it considers how bounded rationality, fragmented political institutions, resistance from concentrated interests, and fiscal constraints usually lead political leaders to adopt incremental policy changes rather than comprehensive reforms even when faced with serious public health problems. | The challenges confronting officials and agencies who are responsible for implementing and administering health policies. Public health professionals who understand the political dimensions of health policy can conduct more realistic research and evaluation, better anticipate opportunities as well as constraints on governmental action, and design more effective policies and programs. |
| Panisset et al., 2012 | discusses the relationships between implementation research and knowledge translation and identifies the role of implementation research | Mixed method | The benefits and synergies needed to translate implementation research into action, how implementation research can be used along the entire continuum of the use of evidence to inform policy. It provides specific examples of the use of implementation research in national level programmes | . A number of tested strategies to support the transfer of implementation research results into policy-making are provided to help meet the standards that are increasingly expected from evidence-informed policy-making practices |
| Uneke et al., 2020 | to describe the process of development and content of the evidence-based policy-making guidance | Systematic review | properly defining/refining policy problem; reviewing contextual issues; initiating policy priority setting; considering political acceptability of policy; commissioning research; use of rapid response services, use of policy advisory/technical/steering committees; and use of policy briefs and policy dialogue. | The strategies to facilitate the use of evidence in policymaking outlined in the Guidance, can be adapted to local context, and incorporated validated approaches that can be used to promote evidence-to-policy-to-practice process |
| Wu et al., 2020 | To examine the role that research plays in influencing health policy | Qualitative study | Research uptake by policy makers influenced by perceived importance of the health issues, relevance of research, government priorities, technical expertise, quality of research, donors influence, financial power and composition of research team. | Resolving conflict of interest between researchers, policymakers, communities is crucial to improve evidence use by policy makers, and understanding the policy making process and making alliance with actors |
| Pucca et al., 2015 | to share part of this experience in order to prompt reflection about the inclusion of oral health care in other health care systems | Historic narrative review | The most significant results of Smiling Brazil can be seen in 3 areas: oral health epidemiological indicators, financial investment and professional development, and the building of an oral health care network throughout the 10 years of the policy. | process undergoing construction and that the oral health care network needs to be further expanded, refined, and solidified so that over time and through changes in the political parties in power |
| Rajabi et al., 2012 | To increase scientific evidence contribution in decision making, evidence-informed health policy making | Review | main audience of the policy are policy makers who order the issue, and capacity building in this field through increasing tendency and motivation in authors and familiarizing policy makers | consider their different audiences by providing specific massages for them in order to promote research knowledge translation |
| Glasziou et al., 2014 | aim to improve the quality of research reports, but all are much less adopted and adhered to than they should be. | Systematic review | studies of published trial reports showed that the poor description of interventions meant that 40-89% were non-replicable; Although best documented in reports of controlled trials, inadequate reporting occurs in all types of studies-animal and other preclinical studies, diagnostic studies, epidemiological studies, clinical prediction research, surveys, and qualitative studies, and understanding of the complex systems | Evidence for some recommendations is clear: change the current system of research rewards and regulations to encourage better and more complete reporting, and fund the development and maintenance of infrastructure to support better reporting, linkage, and warrants future investment in the monitoring of and research into reporting of research, and active implementation |
| Créquit et al., 2016 | aimed to quantify the waste of research related to the failure of systematic reviews to provide a complete and up-to-date evidence synthesis over time | Systematic review | the evidence covered by existing systematic reviews was consistently incomplete: 45 % to 70 % of trials; 30 % to 58 % of patients; 40 % to 66 % of treatments; and 38 % to 71 % of comparisons were missing. In the cumulative networks of randomized evidence, 10 % to 17 % of treatment comparisons were partially covered by systematic reviews and 55 % to 85 % were partially or not covered | illustrate how systematic reviews of a given condition provide a fragmented, out-of-date panorama of the evidence for all treatments. This waste of research might be reduced by the development of live cumulative network meta-analyses. |
| Hamalainen et al., 2015 | The aims of the present article are to explore the use of research evidence in health-enhancing physical activity | Mixed method study | . The evidence used in HEPA policies was found to fall into the following categories: societal framework, media, everyday knowledge and intuition, research evidence, and other types of evidence. | understand their role in translating research evidence into policymaking processes. Research evidence seems to be the only type of evidence used in policymaking. |
| Peirson et al., 2012 | to explore and describe critical factors and dynamics in the early implementation of one public health unit's strategic initiative to develop capacity to make EIDM standard practice. | Qualitative study | Barriers linked to evidence -informed decisionmaking are dynamics for building capacity: clear vision and strong leadership, workforce and skills development, ability to access research, fiscal investments, acquisition and technological resources, a knowledge management, effective communication, a receptive culture, and a focus on change management. | Suggested leadership, planning, commitment and substantial investments, a public health department has made significant progress, within the first two years of a 10-year initiative, towards achieving its goal of becoming evidence informed decision-making organization. |
| Orton et al., 2011 | to synthesis empirical evidence on the use of research evidence by public health decision makers in settings with universal health care systems | Systematic review | Barriers to the use of research evidence included: decision makers' perceptions of research evidence; the gulf between researchers and decision makers; the culture of decision making; competing influences on decision making; and practical constraints. Research targeted at the needs of decision makers; research clearly highlighting key messages; and capacity building. | more effectively implement research informed public health policy, action is required by decision makers and researchers to address the barriers identified. An urgent need for evidence to support the use of research evidence to inform public health decision making to reduce inequalities. |
| Gough et al., 2018 | to provide an overall analysis and description of the what the policy unit works | Narrative review | building a more robust and comprehensive evidence base; raising awareness and understanding regarding the need for using evidence, and; influencing local and national policy to consider evidence more effectively. | the extent and manner of their work outside of these key functions; and their wider strategies to engage and influence their audiences. |
| Franzen et al., 2017 | To explores national stakeholders’ perceptions of processes informing CPG development for primary healthcare | Qualitative study | stakeholder consultation; transparency; management of interests; (communication/co-ordination between CPG development groups; and fit-for-context. Many challenges were attributed to inadequate financial and human resources, which were perceived to hinder capacity to undertake the necessary methodological work, respond to stakeholders’ feedback, and document and share decision-making processes. Challenges were also linked to a complex web of politics, power and interests. | strong commitment amongst national stakeholders to advance CPG development processes, a mix of values, politics, power and capacity constraints pose significant challenges. Contrasting perspectives regarding managing interests and how best to adapt to within-country contexts requires further exploration. |
| Narasimhan et al., 2021 | To explore uptake and scale-up of self-care interventions for sexual and reproductive health and rights | Review | For policy-makers, self-care interventions are important for responding to people’s priorities, needs and rights with regard to health. Ensuring availability of quality, regulated self-care interventions is part of the duty of policy-makers to protect their constituencies against harmful or exploitative practices. | Despite the diversity of the countries in the EMR, the opportunities and challenges in the regional examples show the need for quality data to inform how the public health |
| Tilahun et al., 2015 | To outline recommendations to inform evidence-informed health policymaking in Ethiopia | Review | build capacity for policy analysis and evidence use in creating health policy. Developing functional knowledge management will facilitate using existing evidence, and help with understanding the current scope and state of research. This is essential, but it will not facilitate evidence use on its own. Creating a Health Policy Analysis Unit (PAU) is key. The institutional arrangements of the PAU are critical to ensuring establishment of a capable unit that proactively generates and reviews evidence and health policy. | creating this unit as an independent institute. Inclusive research coordination and priority setting mechanisms will help to meet the research needs, and improve resource use. Using existing mechanisms to more effectively link universities and training programs within universities, engaging academics in existing programs and forums, and promoting academicians to take positions are some important means to improve academic – policymaker links. |
| Schmitz et al., 2016 | To assess how network members have not only contributed to greater global awareness | Review | The analysis reveals a need to transform the network into a formal coalition of regional and national organizations that represent a broader variety of constituents, including the medical community, consumer groups and development-focused non-governmental organizations. | the availability of proven and cost-effective public health interventions, alcohol control represents an excellent ‘buy’ for donors interested. promoting road safety and reducing domestic violence and health care costs caused by alcohol consumption |
| Parkhurst et al, 2018 | explores the importance of both political contestation and institutional context to understand when and how evidence will be used within policy processes. | Review | Political and institutional factors shape the form of evidence use arising in health policy processes. The chapter syntheses insights about how political contestation, issue construction, and institutional arrangements all work (and at times work together) to shape and direct evidence use. | The chapter, however, concludes by recognizing that the insights from this volume only present a starting point to understanding the politics of evidence use from a public policy perspective, merely scratching the surface of the many areas of research that can further be done in this field. |
| Grobbelaar, 2013 | To build capacity for Research Uptake (RU) and its management in two ways, | Review | identified key areas where capacity for knowledge utilization at institutional and systems levels can be built. This framework was developed for the healthcare sector, but the structure has proved useful in the university context as well, as it could be elsewhere | Five areas are identified for “linking research efforts with action; creating a climate for Research Uptake; enabling push factors; exchange mechanisms; enabling pull factors and monitoring & evaluation |
| Sigudla et al.,2020 | to develop a tailored model to enable the optimal uptake of public health research findings for health care practice and policy development in a low-resource country. | Two step Qualitative study | lack of awareness of research results and champions to lead engagements among research stakeholders. Researchers also fail to align public health research projects to existing local contexts and available resources as well as establish and sustain beneficial collaboration between all research stakeholders. Other factors are support, experience, motivation & time factor); research agenda, funding, resources & partnerships), and gatekeeping, local research committees, accessibility of evidence, quality of evidence and critical appraisal skills. | In the context of research uptake for healthcare practice and policy development, we consider this model unique and appropriate for low-resource countries in that it integrated the PARIHS framework with the logical framework to streamline the research uptake process |
| Zhou et al., 2020 | to identify challenges and lessons for LMICs to develop and implement CAMH policy | Systematic review | Six major challenges for research uptake were poor public awareness and low political willingness; stigma against mental disorders; culture values; the lack of CAMH data and evidence; the shortage of CAMH resources, including human resources, service facilities, and funding; and unintended consequence of international support, including reducing local responsibilities, planning fragmentation, and unsustainability. organizations. | Six lessons to overcome challenges were summarized: rethinking the concept of CAMH, encouraging a stand-alone CAMH policy and budget, involving stakeholders, reinforcing the role of research and researchers in policy process, innovating the usage of human and service resources, and maximizing the positive influence of international organizations and non-governmental |
| Conalogue et al., 2017 | to elicit views on the long-term future global health research priorities; areas likely to be less important over time; how to improve research uptake in low-income countries; and build research capacity in LMICs. | Qualitative study | Participants felt that the key area for reducing funding prioritisation was infectious diseases. The involvement of policymakers and other key stakeholders was seen as critical to drive research uptake, as was collaboration and partnership. Several methods to build research capacity in low-income countries were described, including capacity building educational programmes, mentorship programmes and research institution collaboration and partnership. | The outcomes from this survey were reviewed alongside other elements of a wider DfID consultation process to help inform long-term research prioritisation of global health research. There are limitations in this approach; the opportunistic nature of the survey’s dissemination means the findings presented may not be representative of stakeholders or views. |
| Glandon et al., 2018 | to present research priorities on multisectoral collaboration for health from researchers and policymakers around the globe, with an emphasis on LMICs | Mixed method | best to structure, implement and sustain MSCs, as well as how to build stakeholder capacity and community partnerships. Policymakers’ reflections and online ranking by researchers, two topics emerged as research priorities for all three: leadership, partnership and governance structures, implementation strategies and research priority setting mechanisms. | These findings could inform efforts within and beyond the health sector to better align research objectives and funding with the evidence needs of policymakers grappling with questions about how best to leverage MSCs to achieve UHC and the SDGs |
| Brownson et al., 2006 | To assess reasons for systematic translation of evidence to policy | review | Reasons for a lack of consistent and systematic translation of public health research into public policy is examined, including differences in decision-making processes, poor timing, ambiguous findings, the need to balance objectivity and advocacy, personal demands of the process, information overload, lack of relevant data, and the mismatch of randomized thinking with nonrandom problems. | Next, several actions are suggested that should help bridge the chasm between science and policy, such as greater involvement in the process, better understanding of political decision making, building of effective teams, and development of political champions. |
| Oliver et al., 2014 | To identify new barriers of and facilitators to the use of evidence by policymakers, and assess the state of research in this area, we updated a systematic review | Systematic review | The most frequently reported barriers to evidence uptake were poor access to good quality relevant research, and lack of timely research output. The most frequently reported facilitators were collaboration between researchers and policymakers, and improved relationships and skills. There is an increasing amount of research into new models of knowledge transfer, and evaluations of interventions such as knowledge brokerage. | Timely access to good quality and relevant research evidence, collaborations with policymakers and relationship- and skills-building with policymakers are reported to be the most important factors in influencing the use of evidence. Future research and policy priorities should aim to illuminate these concepts and processes, target the factors identified in this review, and consider new methods of overcoming the barriers described. |
